# The kinase PDK1 regulates regulatory T cell survival via controlling redox homeostasis

**DOI:** 10.7150/thno.63992

**Published:** 2021-09-13

**Authors:** Peiran Feng, Quanli Yang, Liang Luo, Yadong Sun, Wenkai Lv, Shuo Wan, Zerong Guan, Zhiqiang Xiao, Feng Liu, Zehua Li, Zhongjun Dong, Meixiang Yang

**Affiliations:** 1 Zhuhai Institute of Translational Medicine, Zhuhai People's Hospital (Zhuhai Hospital Affiliated with Jinan University), Jinan University, Zhuhai, Guangdong, 519000, China.; 2 The Biomedical Translational Research Institute, Faculty of Medical Science, Jinan University, Guangzhou, Guangdong, 510632, China.; 3 School of Medicine and Institute for Immunology, Tsinghua University, Beijing Key Lab for Immunological Research on Chronic Diseases, Tsinghua University, Beijing, 100084, China.

**Keywords:** Treg cell, PDK1, ROS, Iron homeostasis, MAPK, Immune cell death

## Abstract

**Rationale:** Regulatory T cells (Treg cells) play an important role in maintaining peripheral tolerance by suppressing over-activation of effector T cells. The kinase PDK1 plays a pivotal role in conventional T cell development. However, whether PDK1 signaling affects the homeostasis and function of Treg cells remains elusive.

**Methods:** In order to evaluate the role of PDK1 in Treg cells from a genetic perspective, mice carrying the floxed *PDK1* allele were crossbred with *Foxp3*^Cre^ mice to efficiently deleted *PDK1* in Foxp3^+^ Treg cells. Flow cytometry was used to detect the immune cell homeostasis of WT and *PDK1^fl/fl^Foxp3^Cre^* mice. RNA-seq was used to assess the differences in transcriptional expression profile of WT and PDK1-deficient Treg cells. The metabolic profiles of WT and PDK1-deficient Treg cells were tested using the Glycolysis Stress Test and Mito Stress Test Kits by the Seahorse XFe96 Analyser.

**Results:** PDK1 was essential for the establishment and maintenance of Treg cell homeostasis and function. Disruption of PDK1 in Treg cells led to a spontaneous fatal systemic autoimmune disorder and multi-tissue inflammatory damage, accompanied by a reduction in the number and function of Treg cells. The deletion of PDK1 in Treg cells destroyed the iron ion balance through regulating MEK-ERK signaling and CD71 expression, resulting in excessive production of intracellular ROS, which did not depend on the down-regulation of mTORC1 signaling. Inhibition of excessive ROS, activated MEK-Erk signaling or overload Fe^2+^ could partially rescue the survival of PDK1-deficient Treg cells.

**Conclusion:** Our results defined a key finding on the mechanism by which PDK1 regulates Treg cell survival via controlling redox homeostasis.

## Introduction

Regulatory T cells, defined by expression of the transcription factor Foxp3, play a pivotal role in immune tolerance and tissue homeostasis 1, 2. Mutation or absence of the gene encoding Foxp3 rapidly results in the development of multiorgan autoimmune disease 3-5. Conversely, tumors actively recruit Treg cells to limit the anti-tumor immune response and promote angiogenesis and tumor growth 6, 7. The critical role of Treg cells in immune tolerance has led to great interest in identifying the signals that control the homeostasis and function of Treg cells. Several studies have reported the mechanisms involved in the balance between Treg cell proliferation and apoptosis 8, the dependence of Treg cells on paracrine interleukin-2, transcription factors and metabolic regulators 9, 10.

The serine/threonine kinase 3-phosphoinositide-dependent protein kinase 1 (PDK1) is a critical metabolic regulator connecting PI3K and downstream components of mTOR activation 11. PDK1 is important for multiple types of immune cell development and function, including T cells, B cells, and NK cells 12-17. Deletion of PDK1 in T cells via *CD4-Cre* induced chronic inflammation of intestine with a reduction in the number of Treg cells 18. Recently, Yu's group reported that PDK1 is critical for of T follicular helper cell homeostasis 19. However, the regulatory role of PDK1 on Treg cells remains poorly studied.

Reactive oxygen species (ROS) has been shown to be important for T cell differentiation and function 20, 21. However, excessive ROS is detrimental to cell function and survival 22. The ROS produced by the Fenton reaction of excessive iron is an important source of cytotoxic ROS. Iron is essential for a wide range of biological processes, such as DNA synthesis, metabolism, and cell proliferation 23. Cellular iron uptake mainly depends on transferrin receptor protein 1 (TFRC), also known as CD71, which is essential for the maintenance of T cell homeostasis and function. Iron metabolism is also essential for the function of innate immune cells such as macrophages 24. However, the effect of iron homeostasis on the function and survival of Treg cells, especially the regulatory mechanism of iron homeostasis in Treg cells, is largely unknown.

In this study, we found that disruption of PDK1 in Treg cells resulted in early onset of a fatal inflammatory disease, with substantially decreased number of Treg cells. Importantly, we demonstrated that PDK1-deficient Treg cells produced excessive ROS which may be induced via the PDK1-MEK-ERK-CD71 axis, resulting in iron overload in Treg cells. Collectively, our data demonstrated that PDK1 is indispensable for Treg cell survival and function, which is of great significance to immune homeostasis and tolerance.

## Results

### Specific deletion of PDK1 in Treg cells leads to lethal inflammation

**Figure 1:**
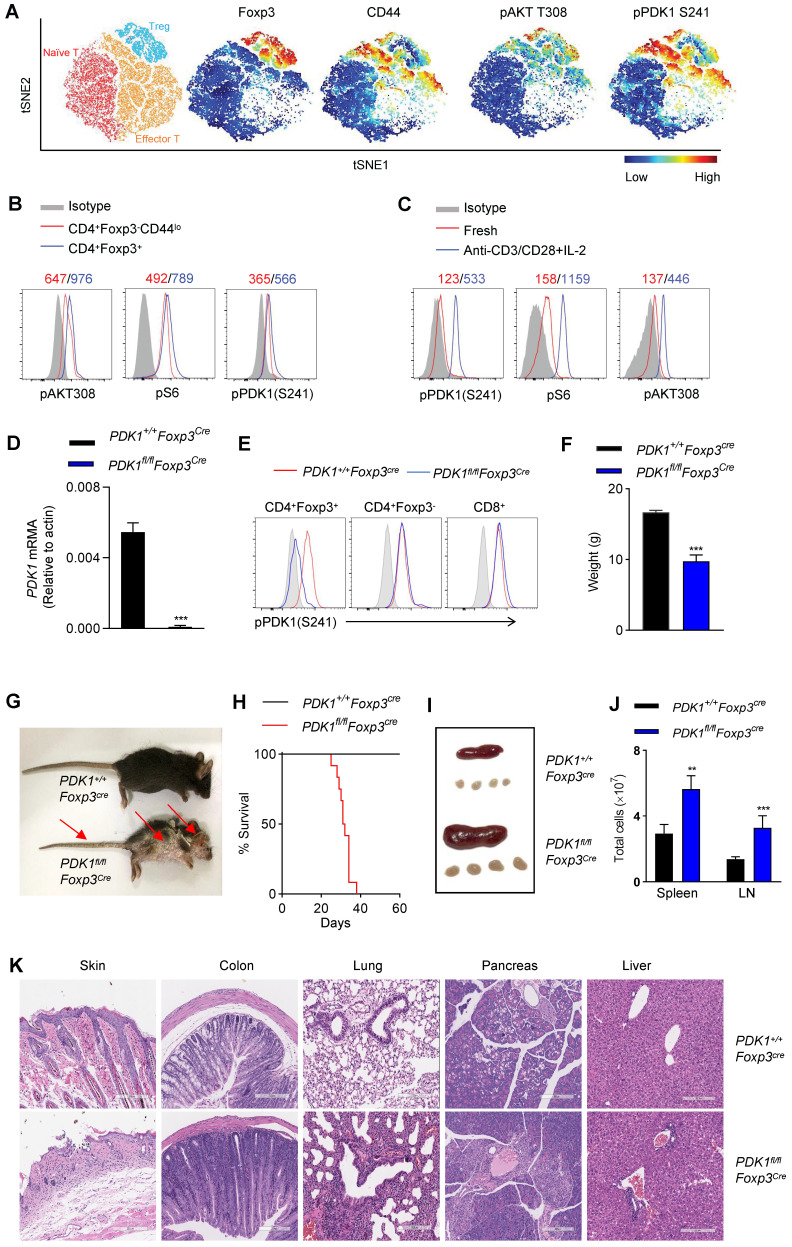
** Treg cells require PDK1 to suppress spontaneous autoimmunity. (A)** Heatmap overlay of Foxp3, CD44, pPDK1 S241, pAKT T308 onto a t-SNE plot generated from splenic CD3^+^CD4^+^ T cells in WT mice. **(B)** Comparison of phosphorylation of PDK1 (S241), S6, and AKT (T308) between naïve T cells (CD4^+^Foxp3^-^CD44^lo^) and Treg cells (CD4^+^Foxp3^+^). **(C)** Comparison of phosphorylation of PDK1 (S241), S6, and AKT (T308) between freshly isolated and activated Treg cells. Mean fluorescent intensity (MFI) is presented above the plot. **(D)** PDK1 gene expression (qPCR) in Treg cells isolated from wild-type and *PDK1^fl/fl^Foxp3^Cre^* mice. **(E)** Analysis of phosphorylation of pPDK1 S241 in CD4^+^Foxp3^+^ Treg cells, CD4^+^Foxp3^-^ T cells and CD8^+^ T cells from the spleen of WT and *PDK1^fl/fl^Foxp3^Cre^*mice. **(F)** Weight of 35-day-old *PDK1^+/+^Foxp3^Cre^* (WT) and *PDK1^fl/fl^Foxp3^Cre^* (KO) mice (n=6). **(G)** Images of 35-day-old *PDK1^+/+^Foxp3^Cre^* and *PDK1^fl/fl^Foxp3^Cre^* mice. Arrows indicate the scaly tail and ulceration of the body. **(H)** Survival curve of *PDK1^+/+^Foxp3^Cre^* and *PDK1^fl/fl^Foxp3^Cre^* mice. (n≥12, P≤0.0001).** (I)** Representative images of spleen and peripheral lymph nodes from *PDK1^+/+^Foxp3^Cre^* and *PDK1^fl/fl^Foxp3^Cre^* mice. **(J)** Cell numbers of the spleen (left) and peripheral lymph nodes (LN) (right) of *PDK1^+/+^Foxp3^Cre^* and *PDK1^fl/fl^Foxp3^Cre^* mice (n≥5). **(K)** Hematoxylin and eosin staining of skin, colon, lung, pancreas, liver (original magnification, ×10), from 35-day-old *PDK1^+/+^Foxp3^Cre^* and *PDK1^fl/fl^Foxp3^Cre^* mice. All results are presented as the mean ± SEM;^ **^P ≤ 0.01; ^***^P ≤ 0.001; unpaired Student's t test. Data represent three independent experiments.

In this study, we aimed to investigate whether the kinase PDK1 regulates Treg cell biology. We firstly examined the amount of phosphorylated PDK1, which represents the activity of PDK1 in naturally occurring Treg cells in a steady state using a multidimensional, unbiased approach. Based on the mapping of the markers such as CD3, CD4, Foxp3, CD44, CD62L, phosphorylated PDK1^S241^ and pAKT^T308^, two-dimensional *t*-distributed stochastic neighbor embedding (*t-*SNE) plots were generated for 3 clusters, including naïve T cells (CD3^+^CD4^+^ Foxp3^-^CD62L^hi^CD44^lo^), effector T cells (CD3^+^CD4^+^ Foxp3^-^CD62L^lo^CD44^hi^) and Treg cells (CD3^+^CD4^+^ Foxp3^+^) (Figure 1A). In fact, the amount of pPDK1^S241^ in Treg cells was relatively higher than that in conventional T cells. Phosphorylation of AKT at Thr 308 and pS6, two representative substrates for PDK1, were also higher expressed in Treg cells (Figure 1A, B). We then examined whether the elevated activity of PDK1 in Treg cells was involved in T-cell activation signaling emanated from TCR plus IL-2 receptor. Indeed, stimulation of Treg cells with anti-CD3/CD28 plus IL-2 significantly increased the amount of phosphorylated PDK1^S241^ and its two substrates, AKT^T308^ and pS6 (Figure 1C). Thus, PDK1 activity is highly preserved in resting Treg cells and prepares for TCR plus IL-2 signaling pathway.

In order to evaluate the role of PDK1 in Treg cells from a genetic perspective, mice carrying the floxed *PDK1* allele were crossbred with *Foxp3*^Cre^ mice. PDK1 was efficiently deleted in Foxp3^+^ Treg cells of *PDK1^fl/fl^Foxp3^Cre^* mice (Figure 1D). Not surprisingly, PDK1 phosphorylation was not detectable in Treg cells lacking PDK1, but it was retained in conventional T cells and CD8^+^ T cells (Figure 1E).

We first noticed that *PDK1^fl/fl^Foxp3^Cre^* mice developed significant weight loss, severe dermatitis with scabs on the ears, eyelids and tail, especially skin ulcers on the head and upper back, along with hair loss, and they eventually died within 6 weeks (Figure 1F-H). *PDK1^fl/fl^Foxp3^Cre^* mice showed splenomegaly and subcutaneous lymphadenopathy with increased cell numbers (Figure 1I, J). The loss of *PDK1* in Treg cells resulted in thymus atrophy with markedly reduced cellularity (Figure S1A-D). Histological analysis of tissue samples from *PDK1^fl/fl^Foxp3^Cre^* mice showed inflammatory cell infiltration in all detected organs (Figure 1K). Together, these phenomena suggest that the absence of PDK1 in Treg cells can lead to early and fatal inflammation.

### Deficiency of PDK1 in Treg cells disrupts immune homeostasis

**Figure 2:**
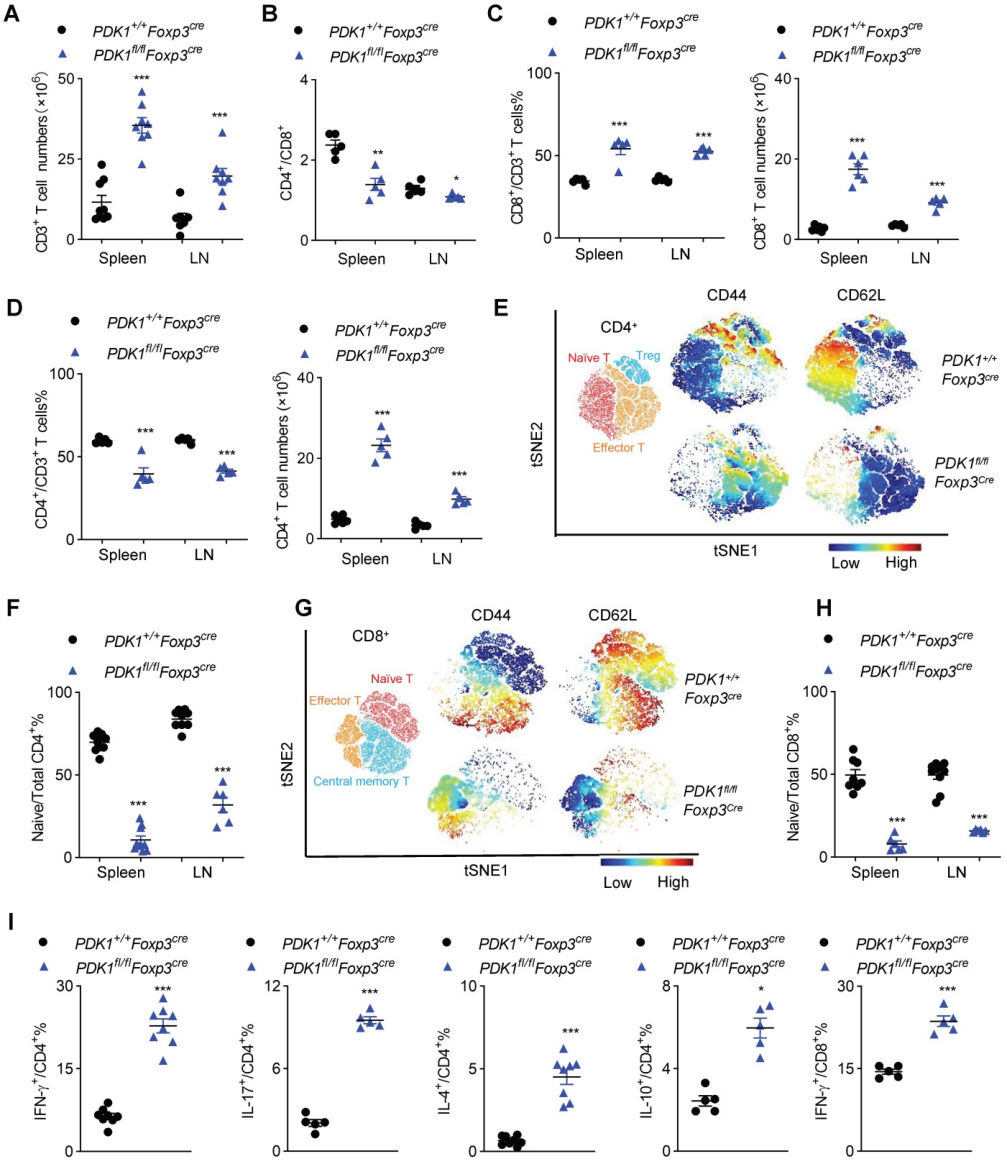
** Disrupted immune homeostasis in *PDK1^fl/fl^Foxp3^cre^* mice. (A)** The number of CD3^+^ T cells in spleen and lymph nodes (LN) from *PDK1^+/+^Foxp3^Cre^* and *PDK1^fl/fl^Foxp3^Cre^* mice (n≥7).** (B)** The ratios of CD4^+^/CD8^+^ T cells in spleen and lymph nodes (LN) from *PDK1^+/+^Foxp3^Cre^* and *PDK1^fl/fl^Foxp3^Cre^* mice (n=5). **(C)** CD3^+^CD8^+^ T cells percentages (left) and numbers (right) in spleen and lymph nodes (LN) from *PDK1^+/+^Foxp3^Cre^* and *PDK1^fl/fl^Foxp3^Cre^* mice (n≥5). **(D)** CD3^+^CD4^+^ T cells percentages (left) and numbers (right) in spleen and lymph nodes (LN) from *PDK1^+/+^Foxp3^Cre^* and *PDK1^fl/fl^Foxp3^Cre^* mice (n=5). **(E)** Heatmap overlay of CD44, CD62L onto a t-SNE plot generated from splenic CD4^+^ T cells in *PDK1^+/+^Foxp3^Cre^* and *PDK1^fl/fl^Foxp3^Cre^* mice. **(F)** Percentage of naïve CD4^+^ T cells in total CD4^+^ T cells in spleen and lymph nodes (LN) from *PDK1^+/+^Foxp3^Cre^* and *PDK1^fl/fl^Foxp3^Cre^* mice (n≥6).** (G)** Heatmap overlay of CD44, CD62L onto a t-SNE plot generated from splenic CD8^+^ T cells in *PDK1^+/+^Foxp3^Cre^* and *PDK1^fl/fl^Foxp3^Cre^* mice. **(H)** Percentage of naïve CD8^+^ T cells in total CD8^+^ T cells in spleen and lymph nodes (LN) from *PDK1^+/+^Foxp3^Cre^* and *PDK1^fl/fl^Foxp3^Cre^* mice (n≥6).** (I)** IFN-γ, IL-17, IL-4 and IL-10 production in CD4^+^ cells and IFN-γ production in CD8^+^ cells from *PDK1^+/+^Foxp3^Cre^* and *PDK1^fl/fl^Foxp3^Cre^* mice (n≥3). All results are presented as the mean ± SEM; ^*^P ≤ 0.05; ^**^P ≤ 0.01; ^***^P ≤ 0.001; unpaired Student's t test. Data represent three independent experiments.

Next, we investigated inflammation-related cellular immunity in *PDK1^fl/fl^Foxp3^Cre^* mice. These mice had more T cells in their spleen and the subcutaneous lymph nodes (Figure 2A). Among T cells, the proportion of CD8^+^ T cells increased, while CD4^+^ T cell decrease dramatically, resulting in a change in the ratio of CD4^+^/CD8^+^ in *PDK1^fl/fl^Foxp3^Cre^* mice. Compared with *Foxp3^Cre^* mice, the number of CD4^+^ and CD8^+^ T cells were significantly increased in *PDK1^fl/fl^Foxp3^Cre^* mice (Figure 2B-D). T cells in *PDK1^fl/fl^Foxp3^Cre^* mice were enlarged, and Ki-67 was highly expressed (Figure S2A). These cells were also highly expressed with more nutrient receptors, such as CD71 (the transferrin receptor) and CD98 (a subunit of L-amino acid transporter) (Figure S2B). These data suggest that T cells in *PDK1^fl/fl^Foxp3^Cre^* mice are highly metabolic and therefore robust proliferating.

To further characterize T cell subsets, we found that *PDK1^fl/fl^Foxp3^Cre^* mice had reduced frequencies of CD62L^hi^CD44^lo^ naïve T cells (both CD4^+^ and CD8^+^) but had increased frequencies of CD62L^low^CD44^hi^ memory/effector T cell in the detected spleen and lymph nodes (Figure 2E-H and Figure S2C, D). Furthermore, CD4^+^ T cells from *PDK1^fl/fl^Foxp3^Cre^* mice produced more pro-inflammatory cytokines such as IFN-γ and IL-17, as well as other cytokines like IL-4 and IL-10. IFN-γ-producing CD8^+^ T cells were also enriched in this genotype (Figure 2I and Figure S2E). Thus, PDK1 deficiency in Treg cells induces T cell subsets to become highly inflammatory.

### PDK1 is necessary for Treg cell differentiation and homeostasis

**Figure 3:**
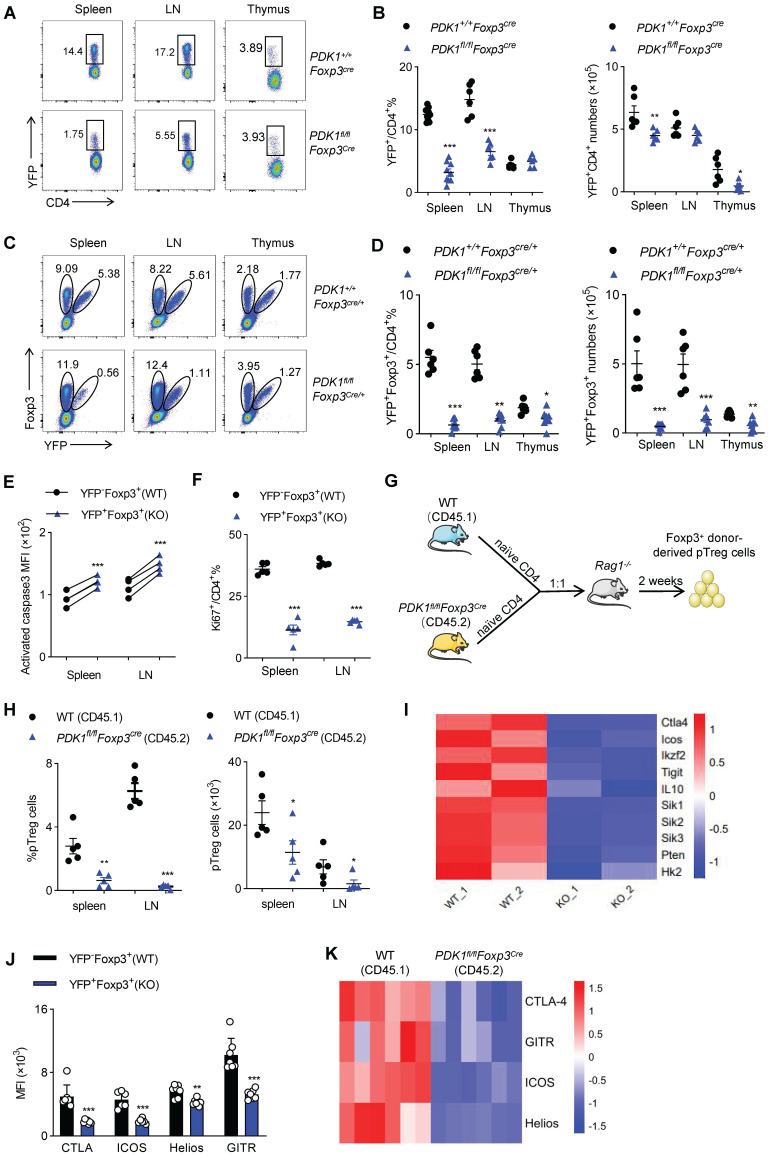
** Treg cell require PDK1 to maintains its survival and proliferation. (A, B)** Representative plots (**A**) and the average frequency (left) and numbers (right) (**B**) of CD4^+^YFP^+^ Treg cells in spleen, lymph nodes (LN) and thymus from *PDK1^+/+^Foxp3^Cre^* and *PDK1^fl/fl^Foxp3^Cre^* mice (3-4 weeks old) (n≥6). **(C)** The fraction of YFP^+^ Treg cells among Foxp3^+^ populations in spleen, lymph nodes (LN) and thymus from heterozygous female *PDK1^+/+^Foxp3^Cre/+^* and *PDK1^fl/fl^Foxp3^Cre/+^* mice, in which half of the Treg compartment expresses YFP-cre. **(D)** Treg cells (Foxp3^+^YFP^+^) percentages (left) and numbers (right) in spleen , lymph nodes (LN) and thymus from *PDK1^+/+^Foxp3^Cre/+^* and *PDK1^fl/fl^Foxp3^Cre/+^* mice (n≥6). **(E)** Expression of Caspase3 in CD4^+^ Foxp3^+^YFP^-^ T cells (WT) and CD4^+^ Foxp3^+^YFP^+^ T cells (KO) in spleen and lymph nodes (LN) from *PDK1^fl/fl^Foxp3^Cre/+^* mice (n=6, paired Student's t test). **(F)** Expression of Ki67 in CD4^+^YFP^+^ T cells in CD4^+^ Foxp3^+^YFP^-^ T cells (WT) and CD4^+^ Foxp3^+^YFP^+^ T cells (KO) in spleen and lymph nodes (LN) from *PDK1^fl/fl^Foxp3^Cre/+^* mice (n≥3, paired Student's t test). **(G)** Experimental schematic for *in vivo* pTreg maintenance assay. **(H)** Percentages (left) and numbers (right) of donor-derived Foxp3^+^ pTreg in *Rag1^-/-^* mice 2 weeks after adoptive co-transfer of naïve CD4^+^ T cells isolated from wild-type (CD45.1) and *PDK1^fl/fl^Foxp3^Cre^* (CD45.2) mice at 1:1 ratio. (paired Student's t test) (n=5). **(I)** Heatmap of differentially expressed genes in Treg cells from *PDK1^+/+^Foxp3^Cre/+^* and *PDK1^fl/fl^Foxp3^Cre/+^* mice. **(J)** MFI statistic of CTLA-4, ICOS, Helios, GITR in CD4^+^ Foxp3^+^YFP^-^ Treg cells (WT) and CD4^+^Foxp3^+^YFP^+^ Treg cells (KO) from *PDK1^fl/fl^Foxp3^Cre/+^* mice. (n=7) (paired Student's t test). **(K)** Heatmap of expression of CTLA4, GITR, ICOS, Helios of donor-derived Foxp3^+^ pTreg in *Rag1^-/-^* mice 2 weeks after adoptive co-transfer of naïve CD4^+^ T cells isolated from wild-type (CD45.1) and *PDK1^fl/fl^Foxp3^Cre^* (CD45.2) mice at 1:1 ratio (n=6). All results are presented as the mean ± SEM; ^*^P ≤ 0.05; ^**^P ≤ 0.01; ^***^P ≤ 0.001; unpaired Student's t test. Data represent two independent experiments.

Next, we figured out whether the fetal inflammation in *PDK1^fl/fl^Foxp3^Cre^* mice was caused by the imbalance in Treg cell number or its function. Compared with control *Foxp3^Cre^* mice,* PDK1^fl/fl^Foxp3^Cre^* mice had much lower proportion of YFP^+^/ Foxp3^+^ Treg cells among CD4^+^ T cells in the detected organs (Figure 3A, B and Figure S3A, B). The decrease in the number of Treg cells might not be due to the change in Foxp3 expression level, because the remaining YFP^+^ Treg cells expressed with a normal amount of Foxp3 (Figure S3C). To rule out the possibility that severe inflammation in *PDK1^fl/fl^Foxp3^Cre^* male mice would induce Treg cell activation phenotype, we took advantage of *PDK1^fl/fl^Foxp3^Cre/+^* female mice, where Cre only expressed in half of the Treg cells due to X-chromosome random inactivation. In theory, the half of Treg cells that are YFP^+^ don't contain PDK1, while the other half Treg cells that are YFP-negative have normal PDK1 expression in *PDK1^fl/fl^Foxp3^Cre/+^* mice. Intriguingly, *PDK1^fl/fl^Fxop3^Cre/+^* mice did not develop any symptoms of inflammation disease. Histological analysis of tissue samples from *PDK1^fl/fl^Foxp3^Cre/+^* mice also showed that there was no inflammatory cell infiltration in the detected organs (Figure S4A). The abnormal ratio of CD4^+^/CD8^+^ and inflammatory T cell subsets observed *in PDK1^fl/fl^Foxp3^Cre^* were never observed in *PDK1^fl/fl^Foxp3^Cre/+^* mice (Figure S4B-D). Therefore,* PDK1^fl/fl^Foxp3^Cre/+^* mice are ideal models to investigate whether severe inflammation in *PDK1^fl/fl^Foxp3^Cre^* in turn interferes with Treg cell homeostasis. Compared with the *PDK1^+/+^Foxp3^Cre/+^* mice, the proportion of YFP-negative Foxp3^+^ Treg cells in* PDK1^fl/fl^Foxp3^Cre/+^* mice relatively increased, but the proportion and absolute number of YFP-positive Foxp3^+^ Treg cells dramatically reduced (Figure 3C, D). Notably, we found that as the mice get older, the proportion of YFP^+^ Treg cells declined progressively (Figure S4E). If we compared YFP^+^ with YFP^-^ Treg cells in *PDK1^fl/fl^Foxp3^Cre/+^* mice, we clearly noticed that YFP^+^ cells were much apoptotic and low proliferative (Figure 3E, F and Figure S4F, G). Therefore, although the over-activated phenotype of Treg cells in *PDK1^fl/fl^Foxp3^Cre^* mice may be attributed to external inflammation, PDK1 is necessary for Treg cell differentiation and homeostasis, regardless of whether it is related to the inflammatory environment.

Finally, to verify whether PDK1 is required for inducing and maintaining peripheral Treg (pTreg) cells, naïve YFP^-^CD4^+^ T cells isolated from WT (CD45.1) mice or *PDK1^fl/fl^Foxp3^Cre^* mice (CD45.2) were adoptively co-transferred into *Rag1^-/-^* mice at 1:1 ratio (Figure 3G). In this chimeric experiment, PDK1 was intact in Treg cells derived by CD45.1^+^ cells, while CD45.2^+^ CD4^+^ T cells obtained the expression of Foxp3, which may drive transcription of transgenic Cre and thus delete the floxed *PDK1* allele in Foxp3^+^ pTreg cells. The frequency and the number of Foxp3-negative CD4^+^ T cells that were differentiated from the two donors were comparable (Figure S4H). However, the proportion and number of CD45.2^+^ pTreg cells in spleen and lymph nodes were significantly lower than that of CD45.1^+^ pTreg (Figure 3H and Figure S4I). Thus, PDK1 is also required for pTreg induction and maintenance.

### PDK1 deficiency alters the gene profile of Treg cells

In order to further reveal the mechanism by which PDK1 affects Treg cell differentiation and function, we isolated YFP^+^ Treg cells from female *PDK1^+/+^Foxp3^Cre/+^* and *PDK1^fl/fl^Foxp3^Cre/+^* mice and analyzed their gene expression profiles by RNA-seq. Intriguingly, genes clusters associated with inhibitory function, proliferation and metabolism of Treg cells were significantly down-regulated (Figure 3I). This difference was further verified by flow cytometry, that is, the expression of CTLA-4, ICOS, Helios and GITR was down-regulated in YFP^+^Foxp3^+^ Treg cells from *PDK1^fl/fl^Foxp3^Cre/+^* mice (Figure 3J and Fig S4J). In addition, the expression of these functional molecules in CD45.2-derived pTreg cells was also significantly down-regulated in the chimeric assay, similar to Figure 3J (Figure 3K). Therefore, PDK1 may enhance the suppressive function of Treg cells by regulating the expression of key functional molecules.

### PDK1 deficiency impairs the function of Treg cells

**Figure 4:**
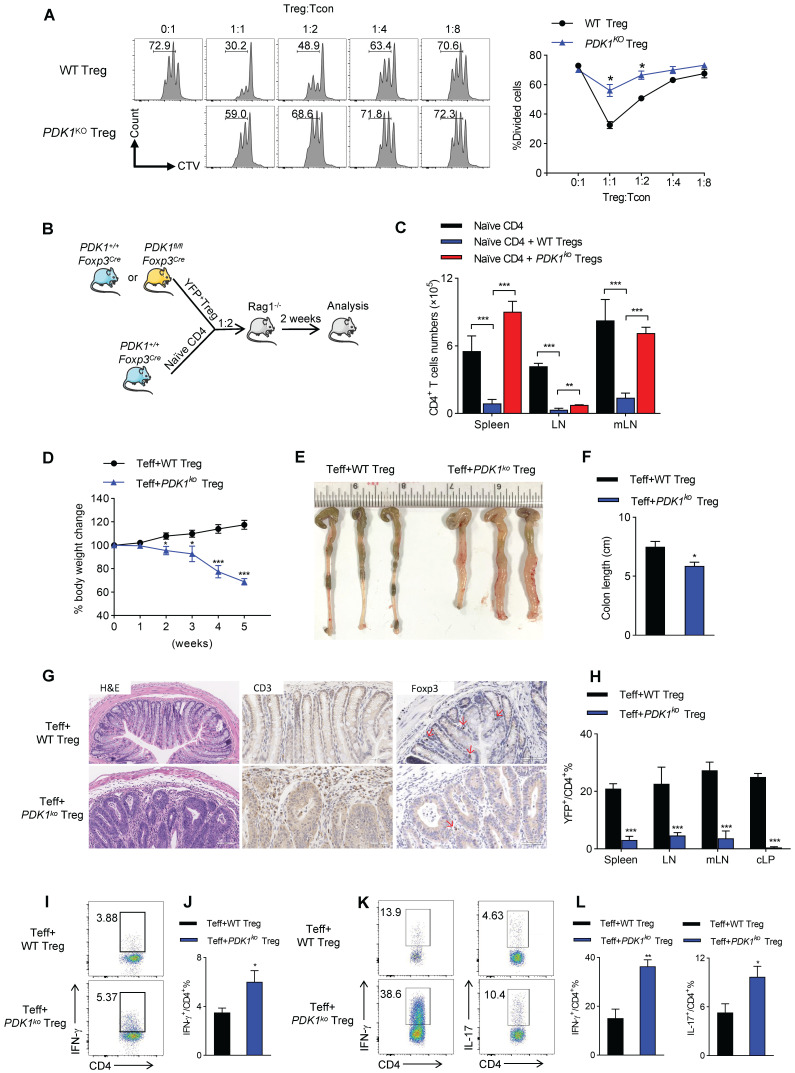
** PDK1 deletion impairs Treg function *in vitro* and *in vivo*. (A)** *In vitro* suppressive activity of Treg cells from *PDK1^+/+^Foxp3^Cre/+^* and *PDK1^fl/fl^Foxp3^Cre/+^* mice. **(B)** Experimental schematic for *in vivo* Treg suppressive activity assay. **(C)** Contrasting effects of WT and PDK1-deficient Tregs on the extent of homeostatic proliferation at 14 days after adoptive transfer of WT naïve CD4^+^ T cells injected into *Rag1^-/-^* mice (n=4). **(D)** Percentage body weight changes over time of *Rag1^-/-^* mice after co-transferred WT or PDK1-deficient (*PDK1^ko^*) Treg cells with WT Teff cells at 1:2 ratio. **(E, F)** Images **(E)** and length **(F)** of colons from *Rag1^-/-^* mice after co-transferred WT or PDK1-deficient Treg cells with WT Teff cells at 1:2 ratio (n≥3). **(G)** Compare the histological morphology, expression of CD3 and Foxp3 of colons from *Rag1^-/-^* mice after co-transferred WT or PDK1-deficient Treg cells with WT Teff cells at 1:2 ratio (original magnification, ×20, ×40, ×40).** (H)** Percentages of Treg cells in spleen, lymph nodes (LN), mesenteric lymph nodes (mLN) and cLP from *Rag1^-/-^* mice after transferred (n=3). **(I, J)** IFN-γ production in CD4^+^ T cells in spleen from *Rag1^-/-^* mice after transferred (n≥3). **(K, L)** IFN-γ and IL-17 production in CD4^+^ T cells in cLP from *Rag1^-/-^* mice after transferred (n≥3). All results are presented as the mean ± SEM; ^*^P ≤ 0.05; ^**^P ≤ 0.01; ^***^P ≤ 0.001; unpaired Student's t test. Data represent three independent experiments.

Based on the gene-profiling (Figure 3I), we then investigated whether PDK1 deficiency would affect the ability of Treg cells to inhibit T cell proliferation and activation. Thus, YFP^+^ Treg cells either from *PDK1^+/+^Foxp3^Cre/+^* or *PDK1^fl/fl^Foxp3^Cre/+^* mice were sorted. In CTV-labeled *in vitro* assay, PDK1-sufficient Treg cells significantly suppressed T cell division, as indicated by CTV dilution. However, even at a condition of 1:1 ratio, PDK1-deficient Treg cells did not prevent T cell division (Figure 4A). This defect was also shown in an *in vivo* assay. Adoptively transferred PDK1-deficient Treg cells did not maintain the homeostasis proliferation of CD4^+^ T cell in *Rag1*-deficient mice (Figure 4B, C). We finally established a classic of T-cell mediated model colitis in *Rag1*-deficeint mice, in which a mixture of CD4^+^CD45RB^+^CD25^-^ effector T cells plus WT or PDK1-deficient Treg cells was transferred. Compared with WT Treg cells as donor, mice receiving PDK1-deleted Treg cells showed weight loss after 2 weeks after transplantation (Figure 4D). Five weeks after cell transfer, the colons of these mice were shortened and thickened (Figure E, F). Histological analysis revealed a loss of goblet cells, a transmural thickening of the colon, and a high infiltration of inflammatory cells. Not surprisingly, histological analysis also showed the colons from mice receiving PDK1-deleted Treg cells infiltrated with higher CD3^+^ T cells, but fewer Foxp3^+^ Treg cells (Figure 4G). Flow cytometric analysis also confirmed that mice receiving PDK1-deleted Treg cells lacked YFP^+^ Treg cells in the spleen, lymph nodes, mesenteric lymph nodes and colon lamina propria (cLP) (Figure 4H), but enriched CD4^+^ T cells producing IFN-γ and IL-17 in spleen (Figure 4I-J) and cLP (Figure 4K-L). Taken together, PDK1 deficiency severely compromises inhibitory function of Treg cells.

### ROS inhibition rescues PDK1-deficiency Treg cell survival

**Figure 5:**
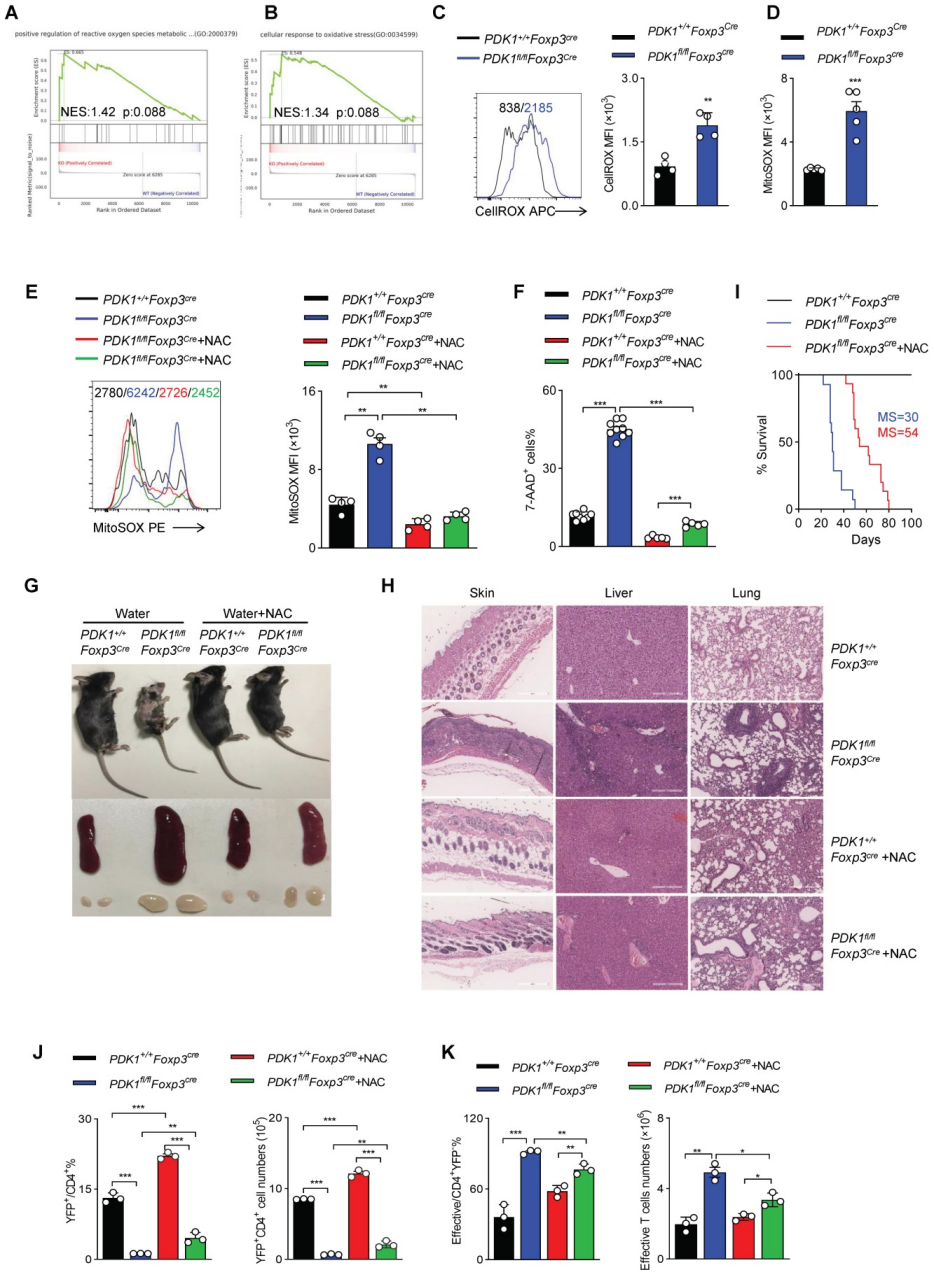
** PDK1 is essential to inhibit excessive ROS-induced Treg cell death. (A, B)** Pathways commonly enriched in WT and PDK1-deficient Treg cells based on GSEA analysis of RNA-seq datasets. **(C)** MFI statistic of total ROS level in Treg cells from *PDK1^+/+^Foxp3^Cre^* and *PDK1^fl/fl^Foxp3^Cre^* mice (n=4). **(D)** MFI statistic of mitochondrial ROS levels in Treg cells from *PDK1^+/+^Foxp3^Cre^* and *PDK1^fl/fl^Foxp3^Cre^* mice (n=5). **(E, F)** Treg cells purified from *PDK1^+/+^Foxp3^Cre^* and *PDK1^fl/fl^Foxp3^Cre^* mice were treated with or without NAC (5mM) for 24h, statistic of mitochondrial ROS levels **(E)** and percentages of 7AAD^+^ cells **(F)** are shown. **(G)** Indicated mice were fed water with or without NAC (1.5g/L) for 20 days from 18-day-old, representative images of mice, spleen and peripheral lymph nodes are shown. **(H)** Hematoxylin and eosin staining of skin, liver, lung, (original magnification, ×10) from indicated mice feed water with or without NAC (1.5 g/L) for 20 days from 18-day-old. **(I)** Survival curve of indicated mice fed water with or without NAC (1.5g/L), median survival (MS) was shown (n≥14). **(J)** The percentage (left) and numbers (right) of Treg cells in spleen from *PDK1^+/+^Foxp3^Cre^* and *PDK1^fl/fl^Foxp3^Cre^* mice fed water with or without NAC (1.5g/L) for 20 days from 18-day-old (n=3). **(K)** The percentage (left) and numbers (right) of effective CD4^+^ T cell in spleen from *PDK1^+/+^Foxp3^Cre^* and *PDK1^fl/fl^Foxp3^Cre^* mice fed water with or without NAC (1.5g/L) for 20 days from 18-day-old (n=3). All results are presented as the mean ± SEM; ^*^P ≤ 0.05; ^**^P ≤ 0.01; ^***^P ≤ 0.001; unpaired Student's t test. Data represent two independent experiments.

To further clarify how PDK1 regulates Treg cell homeostasis, we performed GSEA analysis on RNA-seq datasets containing WT and PDK1-deficient Treg cells. Notably, two sets of ROS-related transcripts, “positive regulation of reactive oxygen species metabolic” and “cellular response to oxidative stress”, were enriched (Figure 5A, B). Thus, the amount of total ROS and mitochondrial ROS in Treg cells was detected. Indeed, compared with WT Treg cells, both total ROS and mitochondrial ROS levels were significantly increased in PDK1-deficient Treg cells (Figure 5C, D). Treg cells of *PDK1^fl/fl^Foxp3^Cre/+^* female mice also produced higher level of ROS than that of control mice (Figure S5A, B). We further investigated whether high level of ROS associated with increased death in PDK1-deleted Treg cells. Mitochondrial ROS level in Treg cells from *PDK1^+/+^Foxp3^Cre^* and *PDK1^fl/fl^Foxp3^Cre^* mice was significantly reduced by NAC (N-acetyl-L-cysteine, used as ROS scavengers) treatment (Figure 5E). Interestingly, NAC treatment could significantly reduce *in vitro* death of Treg cells in both genotypes (Figure 5F). Supplementation of NAC in drinking water for young *PDK1^fl/fl^Foxp3^Cre^* mice could largely reduce the development of inflammation disease, such as less skin ulceration, smaller spleen and lymph nodes and fewer inflammatory cell infiltration in multiple organs after 20 days of post-treatment (Figure 5G, H). Treatment of NAC also significantly extended the lifespan of *PDK1^fl/fl^Foxp3^Cre^* mice likely by increasing the proportion and number of Treg cells (Figure 5I-J). The proportion and number of effective CD4^+^ T cells were also reasonably down-regulated in *PDK1^fl/fl^Foxp3^Cre^* mice after treated with NAC (Figure 5K). Analysis on cytokine showed that CD4^+^ and CD8^+^ T cell IFN-γ secretion were significantly reduced from *PDK1^fl/fl^Foxp3^Cre^* mice after NAC treatment (Figure S5C-F). Thus, ROS-associated cell death impairs the survival of PDK1-deficient Treg cells.

### PDK1 deficiency induces Treg cell apoptosis and iron ion-dependent cell death

**Figure 6:**
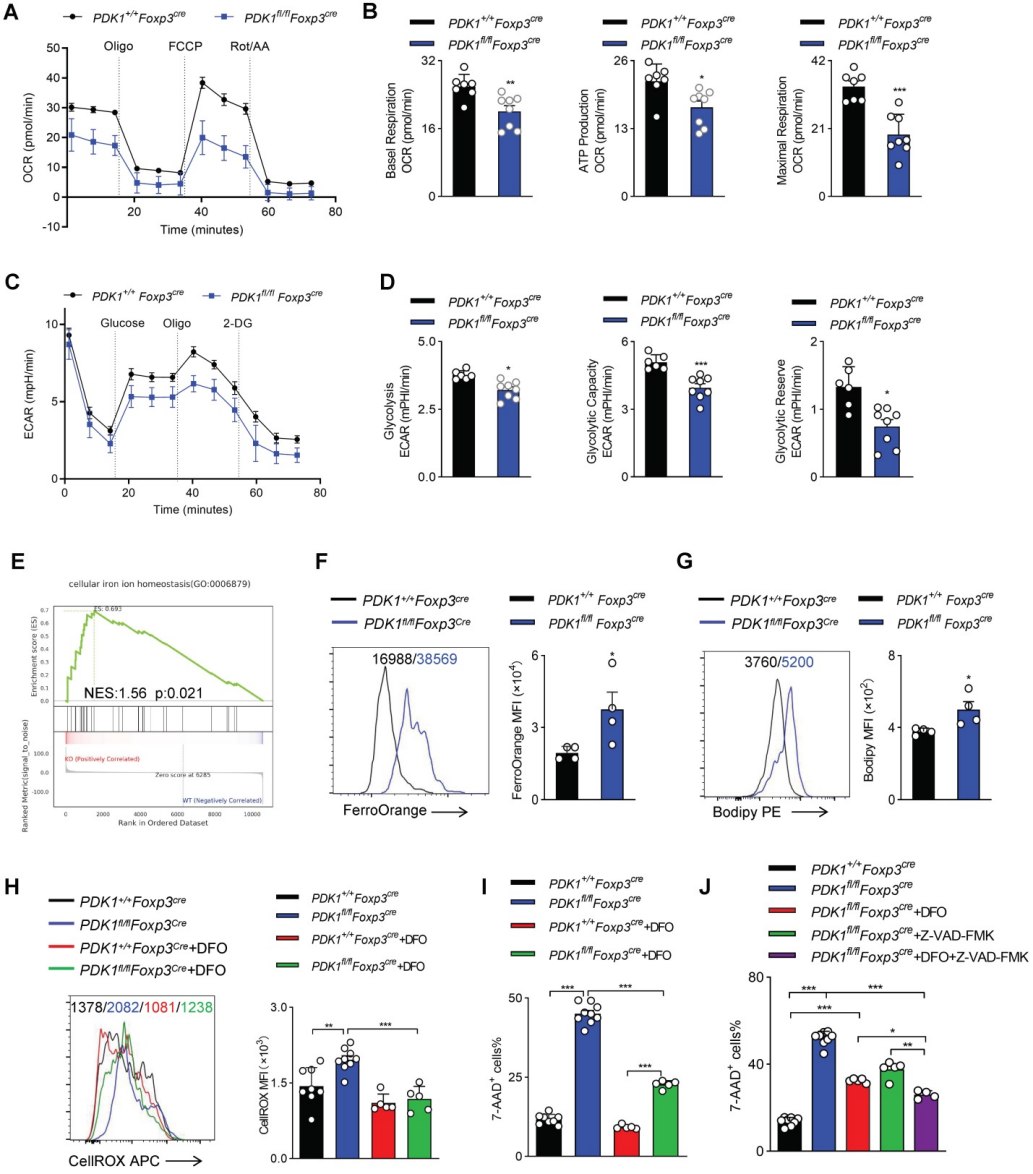
** PDK1 deficiency induces Treg cell apoptosis and iron ion-dependent cell death. (A, B)** OCR analysis of Treg sorted form spleen and lymph nodes (LN) of *PDK1^+/+^Foxp3^Cre^* and *PDK1^fl/fl^Foxp3^Cre^* mice, statistics of 8-9 duplicates were shown in **B**. **(C, D)** ECAR analysis of Treg sorted form spleen and lymph nodes (LN) of *PDK1^+/+^Foxp3^Cre^* and *PDK1^fl/fl^Foxp3^Cre^* mice, statistics of 6-8 duplicates were shown in **D**. **(E)** Pathways commonly enriched in WT and PDK1-deficient Treg cells based on GSEA analysis of RNA-seq datasets. **(F)** MFI statistic of Fe^2+^ level in Treg cells from *PDK1^+/+^Foxp3^Cre^* and *PDK1^fl/fl^Foxp3^Cre^* mice (n=4). **(G)** MFI statistic of Lipid ROS level in Treg cells from *PDK1^+/+^Foxp3^Cre^* and *PDK1^fl/fl^Foxp3^Cre^* mice (n=4).** (H)** Treg cells from *PDK1^+/+^Foxp3^Cre^* and *PDK1^fl/fl^Foxp3^Cre^* mice were treated with or without DFO (200 µM) for 24h, total ROS level was analyzed using CellROX, statistics of 5-8 duplicates were shown. **(I)** Treg cells from *PDK1^+/+^Foxp3^Cre^* and *PDK1^fl/fl^Foxp3^Cre^* mice were treated with or without DFO (200 µM) for 24h, cell viability was analyzed using 7-AAD, statistics of 5-8 duplicates were shown. **(J)** Treg cells from *PDK1^+/+^Foxp3^Cre^* and *PDK1^fl/fl^Foxp3^Cre^* mice were treated with or without DFO (200 µM) or/and Z-VAD (20 µM) for 24h, cell viability was analyzed using 7-AAD, statistics of 4-8 duplicates were shown. All results are presented as the mean ± SEM; ^*^P ≤ 0.05; ^**^P ≤ 0.01; ^***^P ≤ 0.001; unpaired Student's t test. Data represent three independent experiments.

Mitochondria play a key role in regulating cell energy and cell death signal transduction. In addition to producing adenosine triphosphate, mitochondria are also the main source of ROS production 25. To further investigate how ROS excessively generated in PDK1-deficient Treg cells, the mitochondrial functions of Treg cells sorted from* PDK1^+/+^Foxp3^Cre^* and *PDK1^fl/fl^Foxp3^Cre^* mice was assessed by measurement of oxygen consumption rate (OCR). We revealed that the level of OCR was significantly reduced in PDK1-deficient Treg cells (Figure 6A, B). This reduction in OCR level was also shown in the Treg cells of *PDK1^fl/fl^Foxp3^Cre/+^* female mice, which ruled out the effect of inflammatory factors on Treg cell metabolism (Figure S6A, B). Treg cells from both* PDK1^fl/fl^Foxp3^Cre^* and *PDK1^fl/fl^Foxp3^Cre/+^* mice had lower efficiency of ECAR (Figure 6C, D, and Figure S6C, D). These results suggest that PDK1-deficient Treg cells are at a lower metabolic level and the excessive ROS production may not be caused by Treg cell metabolism.

Excess iron ions accelerate lipid catalysis to produce lipid peroxides, which is an important source of cytotoxic ROS in living cells. Interestingly, GSEA analysis suggested an enriched expression of transcript related to “cellular iron ion homeostasis” (Figure 6E). Indeed, the Fe^2+^ level in PDK1-deficient Treg cells was significantly higher than that in control cells (Figure 6F, Figure S6E). Similarly, PDK1 deficiency resulted in increased lipid ROS in Treg cells (Figure 6G, and Figure S6F). In order to evaluate the correlation between excess ROS and iron ion accumulation in Treg cells of *PDK1^fl/fl^Foxp3^Cre^* mice, Treg cells were treated with Deferoxamine (DFO), an iron ion chelator. Intriguingly, DFO treatment significantly reduced total ROS levels and partially rescued the death of PDK1-deficient Treg cells (Figure 6H-I). Therefore, PDK1 deficiency in Treg cells may lead to iron ion homeostasis imbalance, thereby generating excessive lipid ROS. In addition to iron ion-associated cell death, PDK1-deficient Treg cells up-regulated the expression of pro-apoptotic caspase3 (Figure 3E). The apoptosis inhibitor Z-VAD-FMK could partially rescue the death of PDK1-deficient Treg cells (Figure S6G). DFO combined with Z-VAD-FMK could further alleviate the death of PDK1-deficient Treg cells (Figure 6J). However, the cell necrosis inhibitor Nec-1 had no significant effect (Figure S6H). Thus, PDK1 deficiency leads to Treg cell apoptosis and iron ion-dependent cell death, probably ferroptosis.

### PDK1 negatively regulates iron ion-dependent Treg cell death via inhibiting MEK-Erk signaling

**Figure 7:**
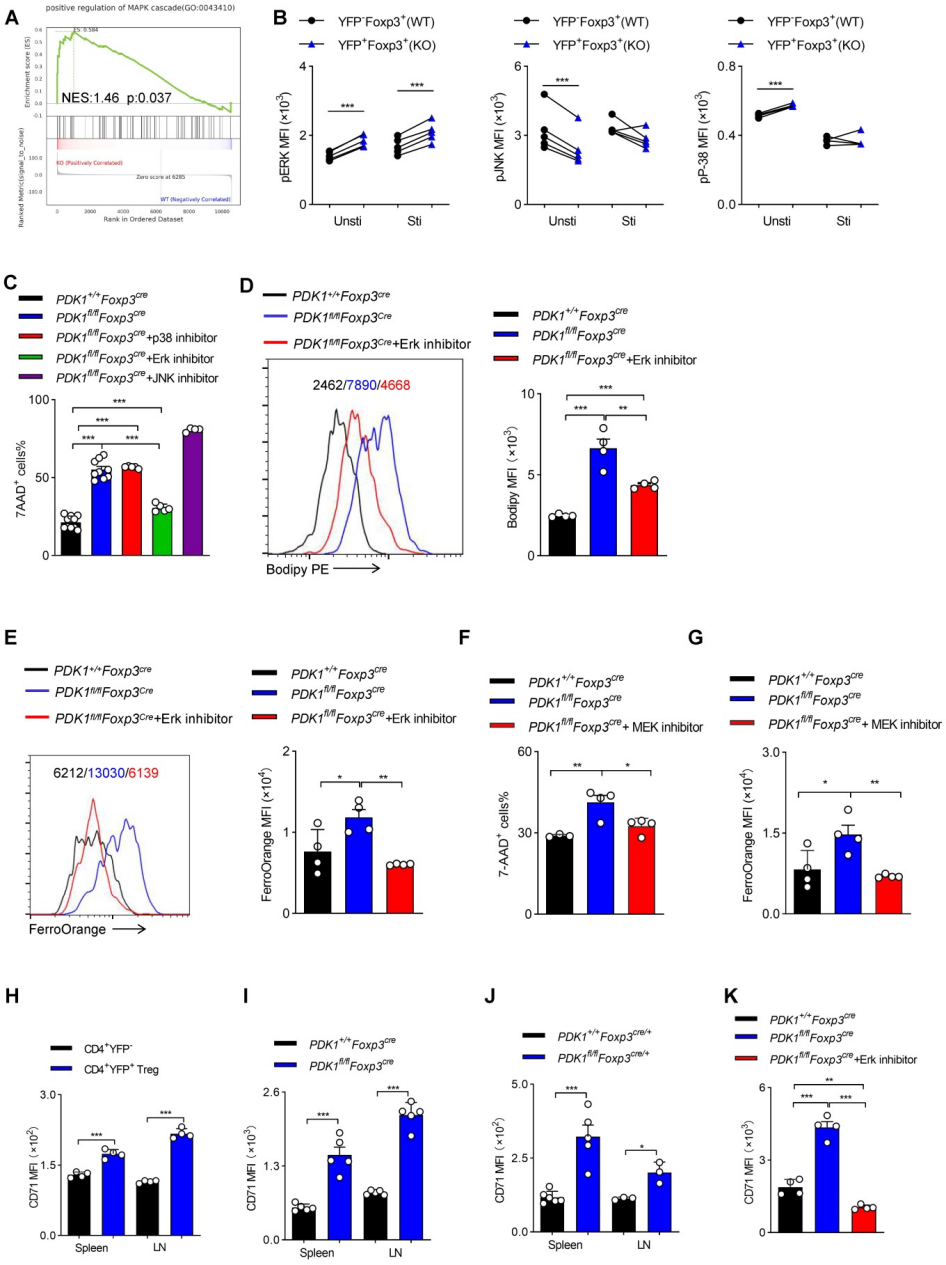
** PDK1 negatively regulates iron ion-dependent Treg cell death via inhibiting MEK-Erk signaling. (A)** Pathways commonly enriched in WT and PDK1-deficient Treg cells based on GSEA analysis of RNA-seq datasets.** (B)** Phosphorylation of Erk1/2 (Thr202/Tyr204), p38 (Thr180/Tyr182) and JNK (Thr183/Tyr185) in CD4^+^ Foxp3^+^YFP^-^ T cells (WT) and CD4^+^ Foxp3^+^YFP^+^ T cells (KO) from *PDK1^fl/fl^Foxp3^Cre/+^* mice freshly detected and activated with anti-CD3/CD28 for 5 min. (paired Student's t test) Unsti: unstimulated, Sti: stimulated (n=5). **(C)** Treg cells from *PDK1^+/+^Foxp3^Cre^* and *PDK1^fl/fl^Foxp3^Cre^* mice were treated with or without p38 inhibitor (SB203580,20 µM), Erk inhibitor (SCH772984,10 µM) and JNK inhibitor (SP600125,20μM) for 24h, cell viability was analyzed using 7-AAD, statistics of 4-10 duplicates were shown. **(D, E)** Treg cells from *PDK1^+/+^Foxp3^Cre^* and *PDK1^fl/fl^Foxp3^Cre^* mice were treated with or without Erk inhibitor (SCH772984,1 µM) for 24h, lipid ROS **(D)** and Fe^2+^ **(E)** level were analyzed by Bodipy 665/676 and FerroOrange, statistics of 4 duplicates were shown. **(F, G)** Treg cells from *PDK1^+/+^Foxp3^Cre^* and *PDK1^fl/fl^Foxp3^Cre^* mice were treated with or without MEK inhibitor (U0126,10 µM) for 24h, cell viability was analyzed using 7-AAD, statistics of 3-4 duplicates were shown in figure **F,** and Fe^2+^ level was analyzed by FerroOrange, statistics of 4 duplicates were shown in figure **G**. **(H)** Comparison of** e**xpression of CD71 between CD4^+^YFP^-^ T cell and CD4^+^YFP^+^ Treg cells in spleen and lymph nodes (LN) from wild type mice (n=4). **(I)** Expression of CD71 in Treg cells from *PDK1^+/+^Foxp3^Cre^* and *PDK1^fl/fl^Foxp3^Cre^* mice (n=5). **(J)** Expression of CD71 in Treg cells in spleen and lymph nodes (LN) from *PDK1^+/+^Foxp3^Cre/+^* and *PDK1^fl/fl^Foxp3^Cre/+^* mice (n≥3). **(K)** Treg cells from *PDK1^+/+^Foxp3^Cre^* and *PDK1^fl/fl^Foxp3^Cre^* mice were treated with or without Erk inhibitor (SCH772984,1 µM) for 24h and the expression of CD71was analyzed, statistics of 4 duplicates were shown. All results are presented as the mean ± SEM; ^*^P ≤ 0.05; ^**^P ≤ 0.01; ^***^P ≤ 0.001; unpaired Student's t test. Data represent three independent experiments.

GSEA analysis suggested that transcripts related to “positive regulation of MAPK cascade” were highly enriched in PDK1-deficient Treg cells (Figure 7A). Ferroptotic death, at least in cancer cells, is usually accompanied by activation of the MAPK signaling pathway. MAPKs family mainly includes p38, ERK and c-Jun NH2-terminal kinase (JNK). PDK1-Akt signal can negatively regulate Raf-MEK-Erk pathway 26. We first verified that in PDK1-deficient Treg cells, only Erk phosphorylation was highly elevated, regardless of resting or activation by TCR stimulation (Figure 7B). The chemical inhibitor for Erk, but not JNK and p38, partially rescued the death of PDK1-deficient Treg cells and reduced lipid peroxides and iron ions accumulation (Figure 7C-E). In the MAPK-Erk pathway, Erk can be activated by MEK 27. The MEK inhibitor also partially enhanced survival of PDK1-deficient Treg cells (Figure 7F) and reduced iron ion production (Figure 7G). Moreover, compared with conventional CD4^+^ T cells, transferrin receptor CD71 was highly expressed on Treg cells (Figure 7H). In particular, CD71 expression in PDK1-deficient Treg cells was much higher than that in WT Treg cells (Figure 7I, J). The Erk inhibitor could significantly inhibit the up-regulation of CD71 in PDK1 deficient Treg cells, thereby preventing intracellular transport of iron ions (Figure 7K). Therefore, PDK1 negatively regulates iron ion-dependent Treg cell death likely probably by inhibiting MEK-Erk signaling and CD71 expression.

PDK1 is the upstream of the mTOR signaling pathway. Previous studies have demonstrated that the mTOR pathway plays an important role in the activation of Treg cells through metabolic reprogramming 1. In addition, Treg cells require mTORC1, rather than mTORC2 signaling to maintain their proliferation and upregulation of the suppressive molecules CTLA4 and ICOS to establish their functional competency 28. We confirmed that mTORC1 signaling was significantly down-regulated in PDK1-deficient Treg cells (Figure S7A). Consistent with previously reports, we showed here that mitochondrial metabolism was inhibited in PDK1-deficient Treg cells. We further found that the ROS level was significantly reduced (Figure S7B), consistent with the results obtained from Raptor^fl/fl^Foxp3^Cre^ mice 28, but the Fe^2+^ level did not change in mTORC1 (*Raptor^fl/fl^CD2^Cre^*)-deficient Treg cells (Figure S7C). These results indicated that the PDK1 maintains iron homeostasis in Treg cells independent of mTORC1 signaling.

## Discussion

In this study, we found that Treg-specific deletion of PDK1 in mice led to development of spontaneous fatal systemic autoimmune disorders, accompanied by reduced Treg number. Treg function assay suggested that PDK1-deficient Treg cells are unable to control effector T cell proliferation and intestinal inflammation. These data indicate that PDK1 plays a critical regulatory role in the survival, proliferation, and suppressive activity of Treg cells.

When we prepared our manuscript, Hyunju Oh *et al.* reported a phenotype similar to ours 29. Based on the high-throughput gene expression analyses, they believe that PDK1 controls Treg cell signature gene expression by regulating the canonical NF-κB pathway. Therefore, we used flow cytometry to detect the activation of the NF-κB signaling in Treg cells from PDK1^fl/^flFoxp3^Cre/+^ mice and found that deletion of PDK1 in Treg cells led to a modest decrease in the expression of pIKKα/β in both physiological state or TCR stimulation condition. In order to explore whether the down-regulation of IKKβ signaling could lead to excessive ROS production, we used SC-514, an IKKβ inhibitor, to treat TCR-stimulated WT Treg cells. We found that both total ROS and mitochondrial ROS in Treg cells of the treatment group were significantly down-regulated (data not shown). These data suggest that the accumulation of ROS in PDK1-deficient Treg cells may not be caused by the down-regulation of pIKKβ. Here, we have elucidated a new mechanism from the perspective of Treg cell redox homeostasis regulation. Specifically, we found that PDK1-deficient Treg cells have enriched expression of two transcripts related to ROS production. Inhibition of excessive ROS could drastically rescue Treg cell survival and alleviate autoimmune diseases, indicating that excessive ROS production was the main reason of PDK1-deficient Treg cell death. As has been known, PDK1 is an important metabolic enzyme that controls cell metabolic fate by regulating mTOR signaling 11. However, our data showed that PDK1-deficient Treg cells were at a lower metabolic level and so unlikely a cause of excessive ROS production. High-throughput gene expression analysis revealed that there was an enriched expression of transcripts related to “Cellular iron ion homeostasis” in PDK1-deficient Treg cells. Both iron deficiency and iron overload can lead to various pathological conditions 23. The maintenance of iron homeostasis is crucial for the adaptive immune system 30-32. The ROS produced by the Fenton reaction of excessive iron is an important source of cytotoxic ROS in living cells. Removing overload of iron ions partially rescued the survival of PDK1-deficient Treg cells, accompanied by downregulation of ROS levels. Therefore, we proved that the activation signals through PDK1 are required for intracellular iron and reactive oxygen species homeostasis in Treg cells.

The increased iron metabolism and lipid peroxidation signaling are recognized as central mediators of ferroptosis 33. Meanwhile, ferroptotic cancer cell death is often accompanied by activation of MAPK signaling pathway. GSEA analysis demonstrated an enriched expression of transcripts related to “positive regulation of MAPK cascade” in PDK1-deficient Treg cells. PDK1-Akt signal can negatively regulate the Raf-MEK-Erk pathway 26. Furthermore, both MEK and Erk inhibitors could partially rescue PDK1 deficiency induced Treg cell death, with reduced lipid peroxides and iron ions. Thus, we identify that PDK1 is important for maintaining iron homeostasis in Treg cells, and so in maintaining Treg cells survival. mTOR signaling is the downstream of PDK1 kinase, the impaired proliferation and the down-regulation of suppressive molecules expression of PDK1-deficient Treg cells may be attributed to the attenuation of mTORC1 signaling. However, the accumulation of iron ions, ROS level were down-regulated in mTORC1-deficient Treg cells, these results suggested that the PDK1 was independent of mTORC1 signaling to maintain Treg cell redox balance.

**Figure 8:**
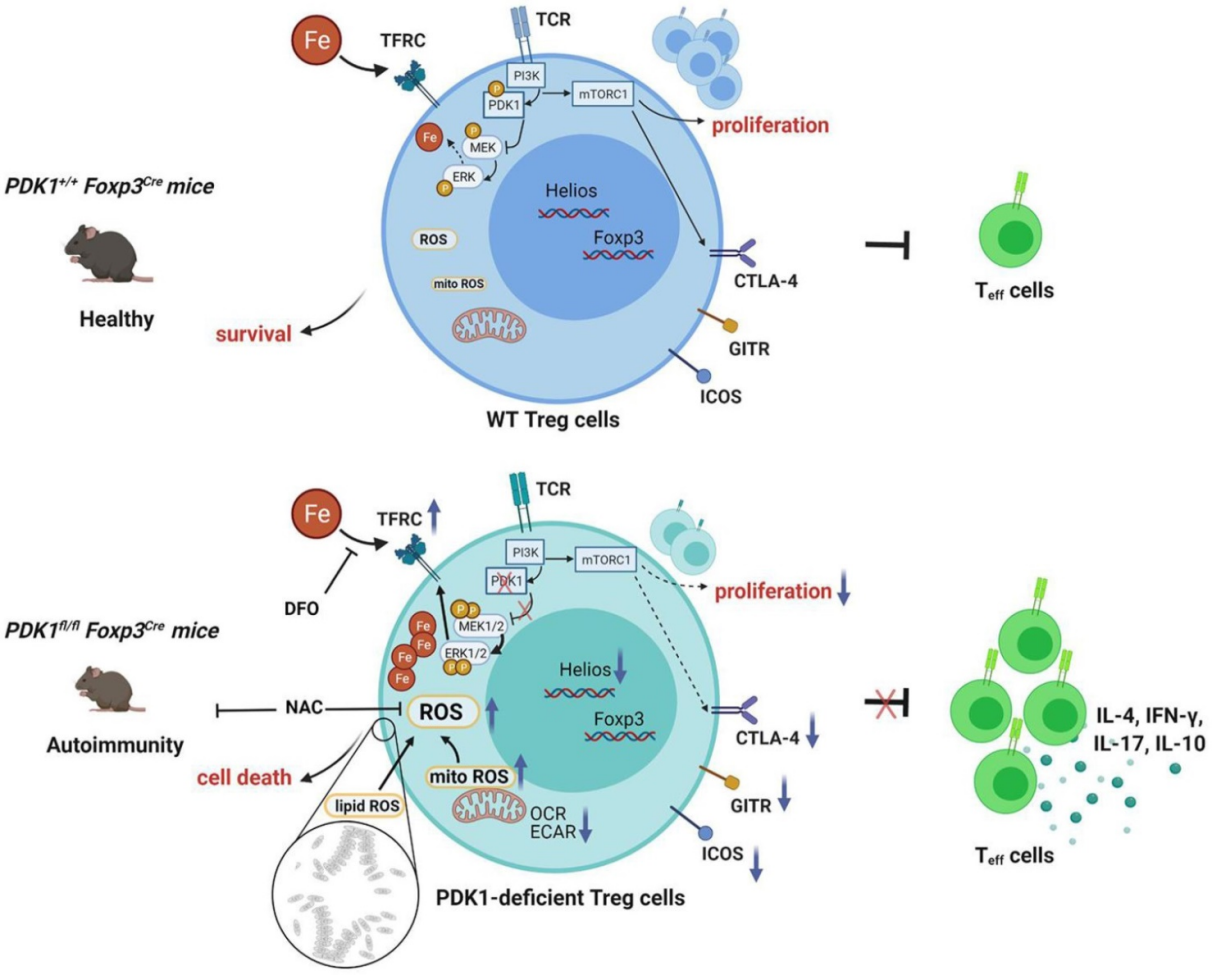
** Schematic illustration of PDK1 regulates regulatory T cell survival via controlling redox homeostasis.** In Treg cells, TCR stimulates PI3K-PDK1-mTOR signaling pathway to maintain the homeostasis and function of Treg cells. Meanwhile, activated PDK1 inhibited the MAPK signaling pathway and maintained the balance of intracellular iron ions. When PDK1 was deleted, the MAPK signaling activity in Treg cells increased, which promoted the up-regulation of the expression of iron transporter CD71, leading to the increase of iron ions in Treg cells, resulting in the production of excessive lipid ROS and the death of Treg cells. In addition, PDK1 also promotes the activation of mTORC1 signaling, which is critical for maintaining Treg cell proliferation and the suppressive molecules expression.

In summary, our study clarifies the effect of PDK1 on the proliferation, survival and function of Treg cells and its potential regulatory mechanism (Figure 8). Additionally, our finding reveals the effect of iron homeostasis on the function and survival of Treg cells, especially the regulatory mechanism of iron homeostasis in Treg cells, which is of great significance to Treg-mediated immune homeostasis and tolerance.

## Materials and methods

### Mice

*PDK1^flox/flox^* mice were constructed in our laboratory. C57BL/6J (B6), *Foxp3^YFP-Cre^*^34^, B6/*Rag1^-/-^* and B6/CD45.1 mice, B6.Cg-Rptor^tm1.1Dmsa^/J (B6 Raptor-flox), and B6.Cg-Tg(CD2-icre)4Kio/J (hCD2-iCre) were purchased from the Jackson Laboratory. *PDK1^flox/flox^* mice were crossed with *Foxp3^YFP-Cre^* mice to generate *PDK1^fl/fl^Foxp3^Cre^* (male) or *PDK1^fl/fl^Foxp3^Cre/+^* (female) mice. *Foxp3^Cre^* (male) or *Foxp3^Cre/+^* (female) mice were used as control. *PDK1^fl/fl^Foxp3^Cre^* mice were used at 3-4 weeks old unless otherwise noted, with the age and gender matched *Foxp3^Cre^* mice as controls. All mice were housed under the specific pathogen-free animal facilities in the Animal Resource Center at Jinan University. All applicable institutional and/or national guidelines for the care and use of animals were followed.

### Flow cytometry

Flow cytometry was performed on BD FACSVerse™ (three-laser flow cytometry analyzer, BD Biosciences). Monoclonal antibodies against mouse CD3 (145-2C11), CD4 (RM4-5), CD44 (1M7), CD62L (MEL-14), Foxp3 (FJK-16s), CD8 (53-6.7), Ki-67 (SolA15), CD98 (RL388), CD71 (R17217), IL-4 (BVD6-24G2), IL-17 (17B7), IL-10 (ES5-16E3), ICOS (7E.17G9), GITR (DTA-1), CTLA-4 (UC10-4B9), Helios (22F6), F(ab) Donkey Anti Rabbit IgG (12-4739-81) and isotype controls were purchased from eBioscience (San Diego, CA). Monoclonal antibodies against mouse anti-GFP (FM264G), IFN-γ (XMG1.2), were purchased Biolegend (San Diego, CA). Monoclonal antibodies against mouse CD25 (7D4), CD45RB (16A), pPDK1 S241 (J66-653.44.17), Caspase-3 (C92-605), 7-AAD (Cat#559925) were purchased BD Biosciences (Mississauga, Ontario, Canada). Anti-phospho-AKT (Thr308) (D25E6), and Anti-phospho-S6 (D57.2.2E), Phospho-p38 MAPK (Thr180/Tyr182) (3D7), Phospho-SAPK/JNK (Thr183/Tyr185) (G9), Phospho-p44/42 MAPK (Erk1/2) (Thr202/Tyr204) (D13.14.4E) were obtained from Cell Signaling Technology (Beverly, MA). Dead cells were excluded from analysis by using LIVE/DEAD fixable Aqua dead cell stain kit (Cat#L34965, Invitrogen) according to the manufacturer's instructions. For analysis of surface markers, cells were stained in PBS containing 2% (w/v) BSA at room temperature. Intracellular staining was performed using Foxp3/transcription factor staining buffer kit (Cat #00-5523-00, eBioscience) according to the manufacturer's instructions. For assessment of cellular and mitochondria ROS in Treg cells, MitoSOX Red (Cat #M36008, Invitrogen) and CellROX Deep Red (Cat #C10422, Invitrogen) were used per the manufacturer's instructions. For assessment of Fe^2+^ level in Treg cells, FerroOrange (Cat #F374, Dojindo) was used the manufacturer's instructions. Flow cytometry data was analyzed using Flowjo software.

### Histology and immunostaining

Tissues were fixed in 4% paraformaldehyde and embedded in paraffin and sliced, followed by hematoxylin and eosin (H&E) staining. The tissue section was scanned by Aperio AT2 (Leica) and analyze with Aperio ImageScope 12.4, a Pathology Slide Viewing Software.

### Cell purification and culture

Lymphocytes were isolated from spleen and peripheral lymph nodes. Total CD4^+^ T cells were bead-isolated used Mouse CD4 T Cell Isolation Kit (Cat#480033, Biolegend). Naïve T cells (CD4^+^YFP^-^CD44^-^CD62L^+^), Treg cells (CD4^+^YFP^+^) cells were sorted on BD FACS AriaII (BD Biosciences). The purified cells were used in the indicated experiments.

### pTreg cell *in vivo* maintenance model

Naïve CD4^+^ T cells (CD4^+^CD25^-^CD44^lo^) were bead-isolated used Mouse CD4 Naïve T Cell Isolation Kit (Cat#480040,Biolegend) from the spleens and peripheral lymph nodes of wild-type (CD45.1) and *PDK1^fl/fl^Foxp3^Cre^* (CD45.2) mice. Then, 5×10^5^ naïve CD4^+^ T cells were transferred via retroorbital injection into *Rag1^-/-^* mice. The presence of Foxp3-YFP^+^ pTreg was evaluated in the spleen and lymph nodes 2 weeks later.

### Treg suppression assays

For *in vitro* studies, 3×10^4^ Treg cells (CD4^+^YFP^+^) cells sorted on BD FACS AriaII (BD Biosciences) from the spleens and peripheral lymph nodes of the respective *PDK1^+/+^Foxp3^Cre/+^* and *PDK1^fl/fl^Foxp3^Cre/+^* mice were co-cultured with Cell Tracker™ Violet (CTV)-labeled naïve T cells (CD4^+^YFP^-^CD44^lo^CD62L^hi^) with anti-CD3 (5 μg/ml), anti-CD28 (1 μg/ml) and IL-2 (2 ng/ml) for 3days. After, CTV-labeled T cell proliferation was determined by flow cytometer, and data was analyzed with FlowJo software. For *in vivo* studies, 1×10^5^ Treg cells (CD4^+^YFP^+^) cells of the respective *PDK1^+/+^Foxp3^Cre^* or *PDK1^fl/fl^Foxp3^Cre^* mice and 2×10^5^ naïve T cells (CD4^+^YFP^-^CD44^lo^CD62L^hi^) of the respective *PDK1^+/+^Foxp3^Cre^* sorted on flow cytometer were co-transferred via retroorbital injection into Rag1^-/-^ mice. Two weeks later, spleen, lymph nodes (LN) and mesenteric lymph nodes (mLN) were harvested and the number of CD4^+^ T cells was determined by flow cytometer and data was analyzed with FlowJo software.

### Adoptive transfer model of colitis

A total of 4×10^5^ Teff cells (CD4^+^CD45RB^hi^CD25^-^) from CD45.1^+^ mice were mixed with 2×10^5^ Treg cells (CD4^+^YFP^+^) from *PDK1^+/+^Foxp3^cre^* mice or *PDK1^fl/fl^Foxp3^cre^* mice, and transferred via retroorbital injection into Rag1^-/-^ mice. Mice were assessed for clinical signs of colitis weekly and analyzed 4-6 weeks after transfer. Colons were harvested for measurement histology which fixed in 4% paraformaldehyde and embedded in paraffin and sliced, followed by hematoxylin and eosin (H&E) staining. Additional sections were stained by immunoperoxidase using primary antibodies directed against CD3 (Cat#GB11014, Servicebio) and FOXP3 (Cat#12653, CST). Lymphocytes isolated from spleen, lymph nodes (LN), mesenteric lymph nodes (mLN) and colon lamina propria (cLP) were analyzed by flow cytometry.

### Metabolic studies

The extracellular acidification rate (ECAR) and oxygen consumption rate (OCR) were measured by the glycolysis stress test kit (Cat#103020-100, Agilent Technology) and mito stress test kit (Cat#103015-100, Agilent Technology), respectively, with a XF96 extracellular flux analyzer (Agilent Technology). Treg cells (CD4^+^YFP^+^) were sorted by flow cytometer from the spleens and peripheral lymph nodes of the respective *PDK1^+/+^Foxp3^Cre^* mice, *PDK1^fl/fl^Foxp3^Cre^* mice, *PDK1^+/+^Foxp3^Cre/+^* and *PDK1^fl/fl^Foxp3^Cre/+^* mice. Treg cells were seeding onto a 96-well XF plate coated with Cell-Tak (Cat# 354240; Corning) with 2×10^5^/well at 37 °C incubator for 30 min and analyzed by XF96 extracellular flux analyzer later. For glycolysis stress test, followed by the sequential addition of glucose (10 mM), oligomycin (1 µM) and 2-DG (50 mM). For mito stress test, Treg cells were followed by the sequential addition of oligomycin (1 µM), FCCP (1.5 µM), Rotenone/antimycin A (0.5 µM). The data was analyzed by Seahorse Wave Desktop v2.4 software.

### RNA isolation and reverse transcriptase PCR

Total RNA was obtained from purified CD4^+^YFP^+^Treg cells from *PDK1^+/+^Foxp3^Cre^* and* PDK1^fl/fl^Foxp3^Cre^* mice and reverse-transcribed with a Takara reverse transcription kit. The primers for the mouse genes were: Pdk1-F: CATACAGACCAGGTTGACAT, Pdk1-R: TGGATATACCTGGACACAGT, the primers for internal control gene GAPDH were: Gapdh-F: CCAGCTTAGGTTCATCAGGT, Gapdh-R: TTGATGGCAACAATCTCCAC. Real-time PCR was performed with a BioRad CFX Connect cycler.

### RNA-seq

Treg cells were sorted from *PDK1^+/+^Foxp3^Cre/+^* (WT) and *PDK1^fl/fl^Foxp3^Cre/+^* (KO) mice, total RNA was extracted using the RNeasy Micro Kit (50) (Qiagen 74004) according to the manufacturer's protocol. RNA purity and quantification were evaluated using the NanoDrop 2000 spectrophotometer (Thermo Scientific, USA). RNA integrity was assessed using the Agilent 2100 Bioanalyzer (Agilent Technologies, Santa Clara, CA, USA). Then the libraries were constructed using Single Cell Full Length mRNA-Amplification Kit (Vazyme, N712-03, Nanjing, China) and TruePrep DNA Library Prep Kit V2 for Illumina (Vazyme, TD502-02, Nanjing, China) according to the manufacturer's instructions. The transcriptome sequencing and analysis were conducted by OE Biotech Co., Ltd. (Shanghai, China). The RNAseq data were deposited in the Sequence Read Archive (SRA) repository at NCBI under the accession number PRJNA704505.

### Statistics

Statistical significance was calculated by two-tailed unpaired Student t test using GraphPad Prism 8 for Windows (GraphPad). The following terminology is used to show statistical significance: *p < 0.05, **p < 0.01, ***p < 0.001. No samples or animals were excluded from the analysis. Variances are similar between the groups in t test analysis.

## Supplementary Material

Supplementary figures.
