# Supplementary material for: The kinase PDK1 regulates regulatory T cell survival via controlling redox homeostasis
Source: Theranostics. 2021 Sep 13;11(19):9503–18. doi: 10.7150/thno.63992 (PMC8490516; doi:10.7150/thno.63992)
Supplement: Supplementary file 1 — Supplementary figures. [file thnov11p9503s1.pdf]

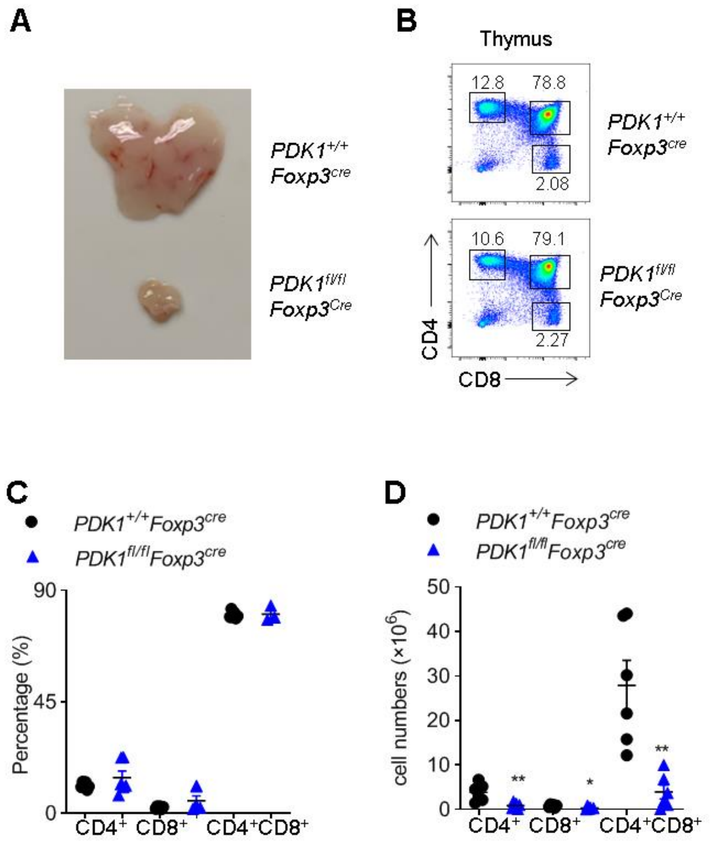

Figure S1. Thymus atrophy in Foxp3^{cre}PDK1$^{fl/fl}$ mice. (**A)** Representative images of thymus from *PDK1$^{+/+}$Foxp3^{Cre}* and *PDK1$^{fl/fl}$Foxp3^{Cre}* mice. (**B)** Thymic CD4^{+} and CD8^{+} T cell populations from *PDK1$^{+/+}$Foxp3^{Cre}* and *PDK1$^{fl/fl}$Foxp3^{Cre}* mice. Thymic CD4^{+} and CD8^{+} T cell percentages (**C**) and numbers (**D**) in thymus from *PDK1$^{+/+}$Foxp3^{Cre}* and *PDK1$^{fl/fl}$Foxp3^{Cre}* mice (n≥3). $^{*}P \leq 0.05$; $^{**}P \leq 0.01$; unpaired Student's t test. Data represent three independent experiments.

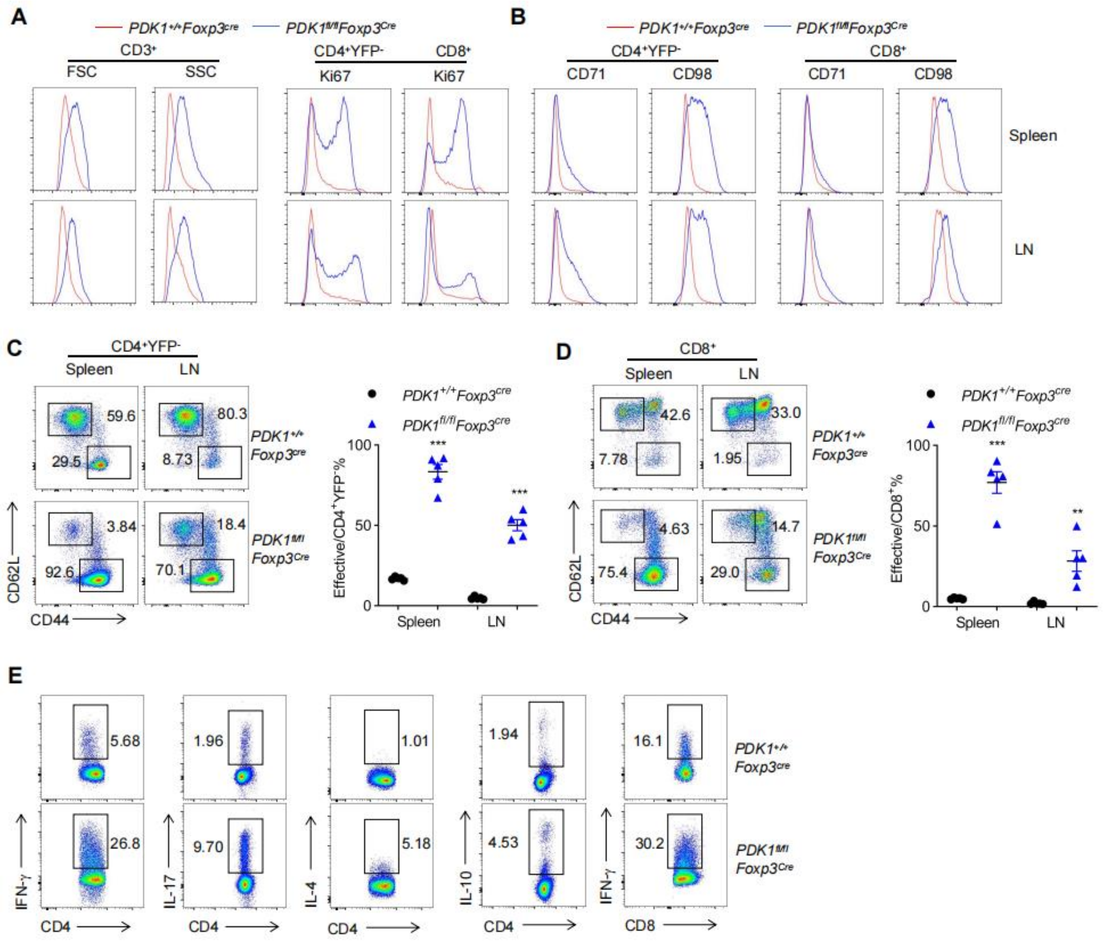

Figure S2. PDK1 deletion in Foxp3$^+$ Tregs caused activation of conventional T cells.

(A) Flow cytometry analysis of the CD3$^+$T cells for the changes of FSC, SSC (left) and expression of Ki67 in CD4$^+$YFP$^-$ T cells and CD8$^+$T cells (right) in spleen and lymph nodes (LN) from *PDK1$^{+/+}$Foxp3^{Cre}* and *PDK1$^{fl/fl}$Foxp3^{Cre}* mice. (**B)** Expression of CD71, CD98 in CD4$^+$YFP$^-$ T cells (left) and CD8$^+$T cells (right) in spleen and lymph nodes (LN) from *PDK1$^{+/+}$Foxp3^{Cre}* and *PDK1$^{fl/fl}$Foxp3^{Cre}* mice. (**C, D)** Left, flow cytometric analysis of the expression of CD62L and CD44 on CD4$^+$YFP$^–$ T cells **(C)** or CD8$^+$ T cells **(D)** from *PDK1$^{+/+}$Foxp3^{Cre}* and *PDK1$^{fl/fl}$Foxp3^{Cre}* mice (n=5). Right, effective (CD62L^{lo}CD44^{hi}) CD4$^+$ T cells **(C)** or CD8$^+$ T cells **(D)** percentage in spleen and lymph nodes (LN). **(E)** Representative image of IFN-γ, IL-17, IL-4 and IL-10

production in CD4^{+} cells and IFN-γ production in CD8^{+} cells from *PDK1*$^{+/+}$*Foxp3*^{Cre} and *PDK1*$^{fl/fl}$*Foxp3*^{Cre} mice. $^{**}P \leq 0.01$; $^{***}P \leq 0.001$; unpaired Student's t test. Data represent three independent experiments.

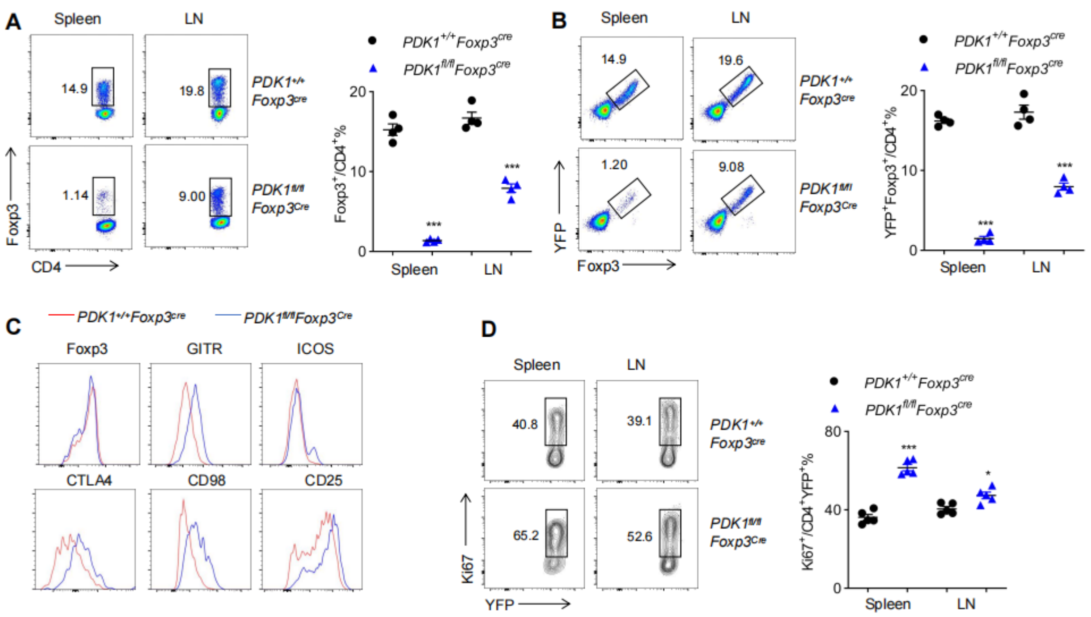

Figure S3. Loss of PDK1 in Foxp3^{+} Treg cells causes decreased frequencies and number of Foxp3^{+} Treg cells. (A) Representative plots (left) and the average frequency (right) of CD4^{+}Foxp3^{+} Treg cells in spleen and lymph nodes (LN) from *PDK1*$^{+/+}$*Foxp3*^{Cre} and *PDK1*$^{fl/fl}$*Foxp3*^{Cre} mice (3-4 weeks old). (n=4). **(B)** Representative plots (left) and the average frequency (right) of CD4^{+}Foxp3^{+}YFP^{+} Treg cells in spleen and lymph nodes (LN) from *PDK1*$^{+/+}$*Foxp3*^{Cre} and *PDK1*$^{fl/fl}$*Foxp3*^{Cre} mice (3-4 weeks old). **(C)** Expression of Foxp3, GITR, ICOS, CTLA4, CD98 and CD25 in CD4^{+}YFP^{+} Treg cells from *PDK1*$^{+/+}$*Foxp3*^{Cre} and *PDK1*$^{fl/fl}$*Foxp3*^{Cre} mice. **(D)** Expression of Ki67 in CD4^{+}YFP^{+} T cells in spleen and lymph nodes (LN) from

PDK1$^{+/+}$*Foxp3*^{Cre} and *PDK1*$^{fl/fl}$*Foxp3*^{Cre} mice (n=5). $^{*}P \leq 0.05$, $^{***}P \leq 0.001$; unpaired Student's t test. Data represent two independent experiments.

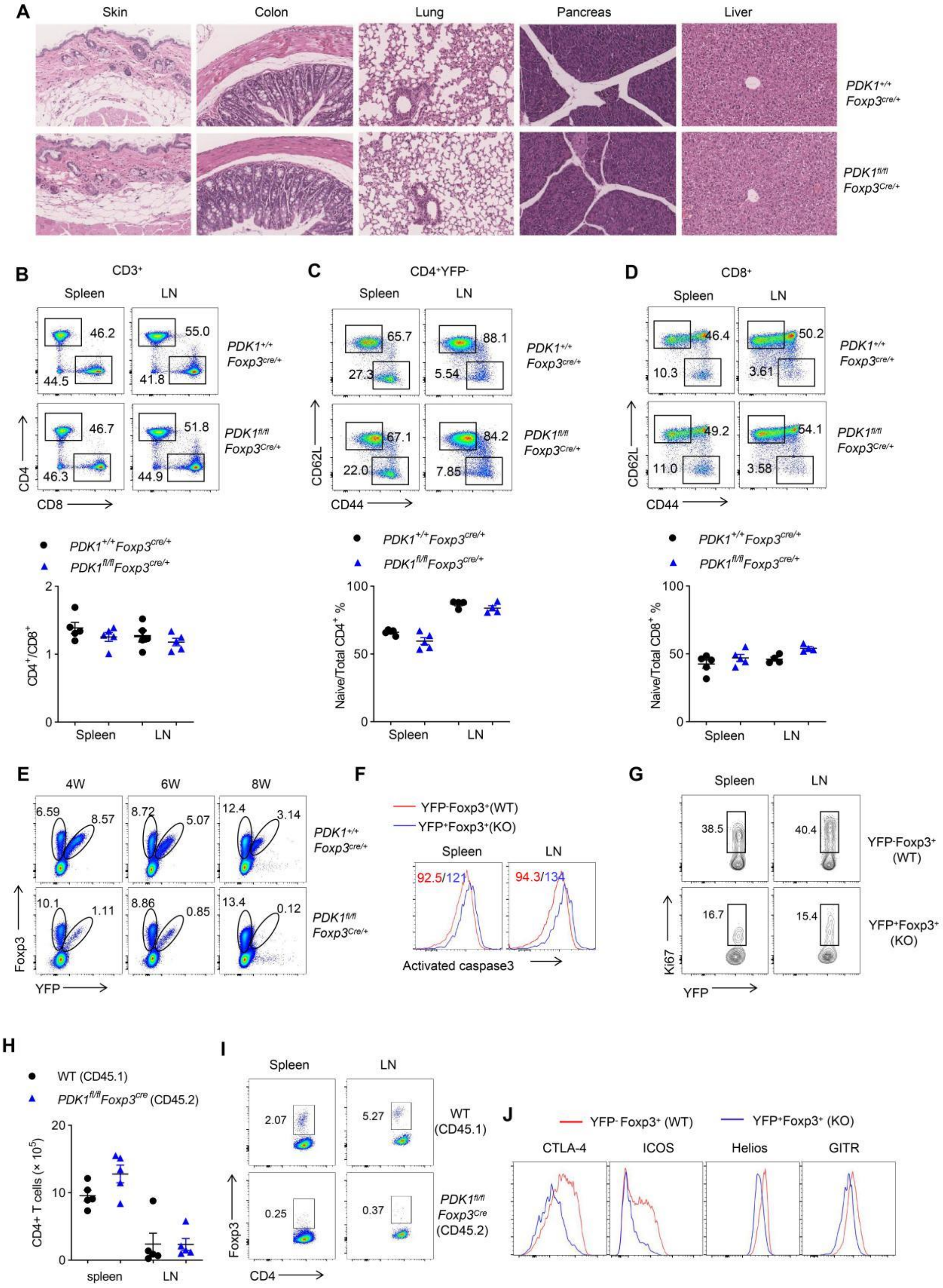

Figure S4. Loss of PDK1 in Foxp3^{+} Treg cells causes decreased frequencies and number of Foxp3^{+} Treg cells under noninflammatory station. **(A)** Haematoxylin and eosin staining of skin, colon, lung, pancreas, liver (original magnification, ×10), from $PDK1^{+/+}Foxp3^{Cre/+}$ and $PDK1^{fl/fl}Foxp3^{Cre/+}$ mice. **(B)** Up, expression of CD4 and CD8 on CD3^{+} T cells in spleen and lymph nodes (LN) from $PDK1^{+/+}Foxp3^{Cre/+}$ and $PDK1^{fl/fl}Foxp3^{Cre/+}$ mice. Down, the ratios of CD4^{+}/CD8^{+} T cells in spleen and lymph nodes (LN) (n=5). **(C)** Up, expression of CD62L and CD44 on CD4^{+}YFP^{-} T cells in spleen and lymph nodes (LN) from $PDK1^{+/+}Foxp3^{Cre/+}$ and $PDK1^{fl/fl}Foxp3^{Cre/+}$ mice. Down, naïve (CD62L^{hi}CD44^{lo}) CD4^{+} T cell percentage in spleen and lymph nodes (LN) (n≥4). **(D)** Up, expression of CD62L and CD44 on CD8^{+} T cells in spleen and lymph nodes (LN) from $PDK1^{+/+}Foxp3^{Cre/+}$ and $PDK1^{fl/fl}Foxp3^{Cre/+}$ mice. Down, naïve (CD62L^{hi}CD44^{lo}) CD8^{+} T cell percentage in spleen and lymph nodes (LN) (n≥4). **(E)** The fraction of YFP^{+} Treg cells among Foxp3^{+} populations in heterozygous female $PDK1^{+/+}Foxp3^{Cre/+}$ and $PDK1^{fl/fl}Foxp3^{Cre/+}$ mice at different age. **(F, G)** Represent plots show the caspase 3 activity **(F)** and Ki67 expression **(G)** in CD4^{+} Foxp3^{+}YFP^{-} Treg cells (WT) and CD4^{+} Foxp3^{+}YFP^{+} Treg cells （KO) from $PDK1^{fl/fl}Foxp3^{Cre/+}$ mice. **(H)** The number of donor-derived CD4^{+} T cells in $Rag1^{-/-}$ mice (n=5). **(I)** Representative plots show the percentage of donor-derived Foxp3^{+} pTreg in $Rag1^{-/-}$ mice 2 weeks after adoptive transfer. **(J)** Represent overlay plots show the expression of CTLA4, ICOS, Helios and GITR in CD4^{+} Foxp3^{+}YFP^{-} Treg cells (WT) and CD4^{+} Foxp3^{+}YFP^{+} Treg cells （KO) from $PDK1^{fl/fl}Foxp3^{Cre/+}$ mice.

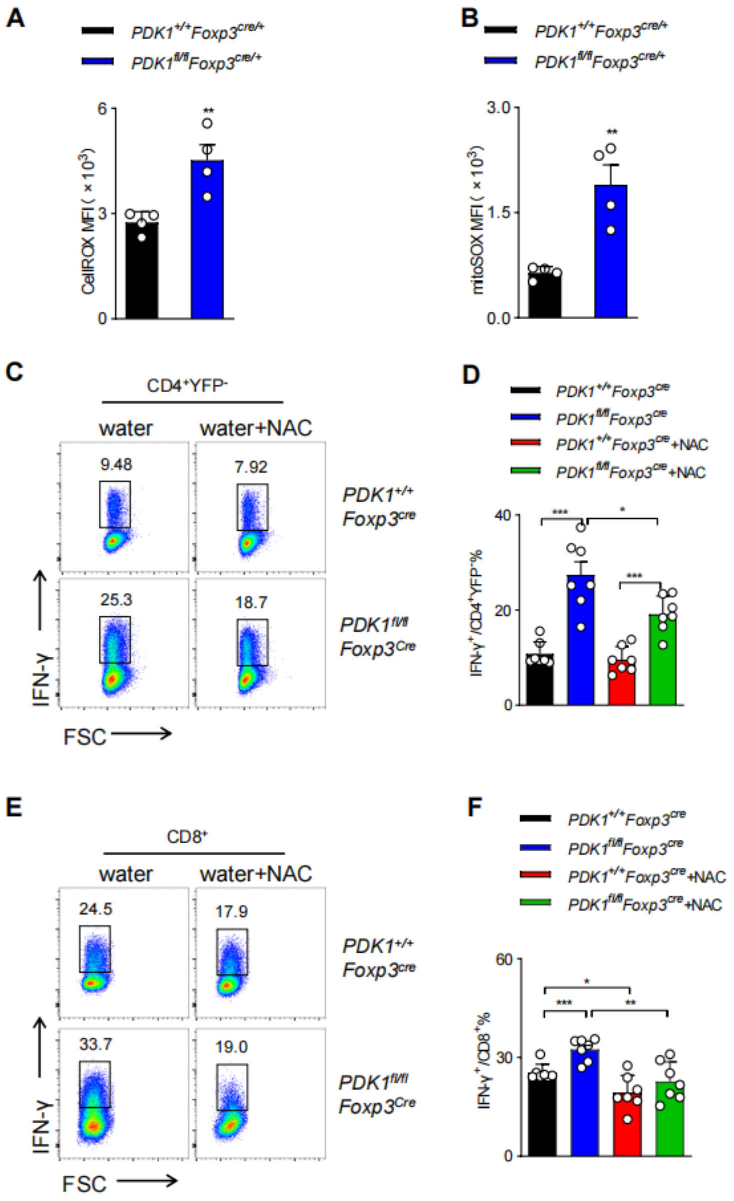

Figure S5. NAC could inhibit the secretion of IFN-γ by effector T cell caused by PDK1 deficiency in Treg cells *in vivo*. (A) MFI statistic of total ROS level in Treg cells from $PDK1^{+/+}Foxp3^{Cre/+}$ and $PDK1^{fl/fl}Foxp3^{Cre/+}$ mice (n=4). **(B)** MFI statistic of mitochondrial ROS level in Treg cells from $PDK1^{+/+}Foxp3^{Cre/+}$ and $PDK1^{fl/fl}Foxp3^{Cre/+}$ mice (n=4). **(C, D)** IFN-γ production in $CD4^{+}YFP^{-}$ T cells in spleen from $PDK1^{+/+}Foxp3^{Cre}$ and $PDK1^{fl/fl}Foxp3^{Cre}$ mice fed water with or without NAC (1.5g/L) for 20 days from 18-day-old (n=7). **(E, F)** IFN-γ production in $CD8^{+}$ T cells in spleen

from $PDK1^{+/+}Foxp3^{Cre}$ and $PDK1^{fl/fl}Foxp3^{Cre}$ mice fed water with or without NAC (1.5g/L) for 20 days from 18-day-old (n=7). $^{*}P \leq 0.05$; $^{**}P \leq 0.01$; $^{***}P \leq 0.001$; unpaired Student's t test. Data represent two independent experiments.

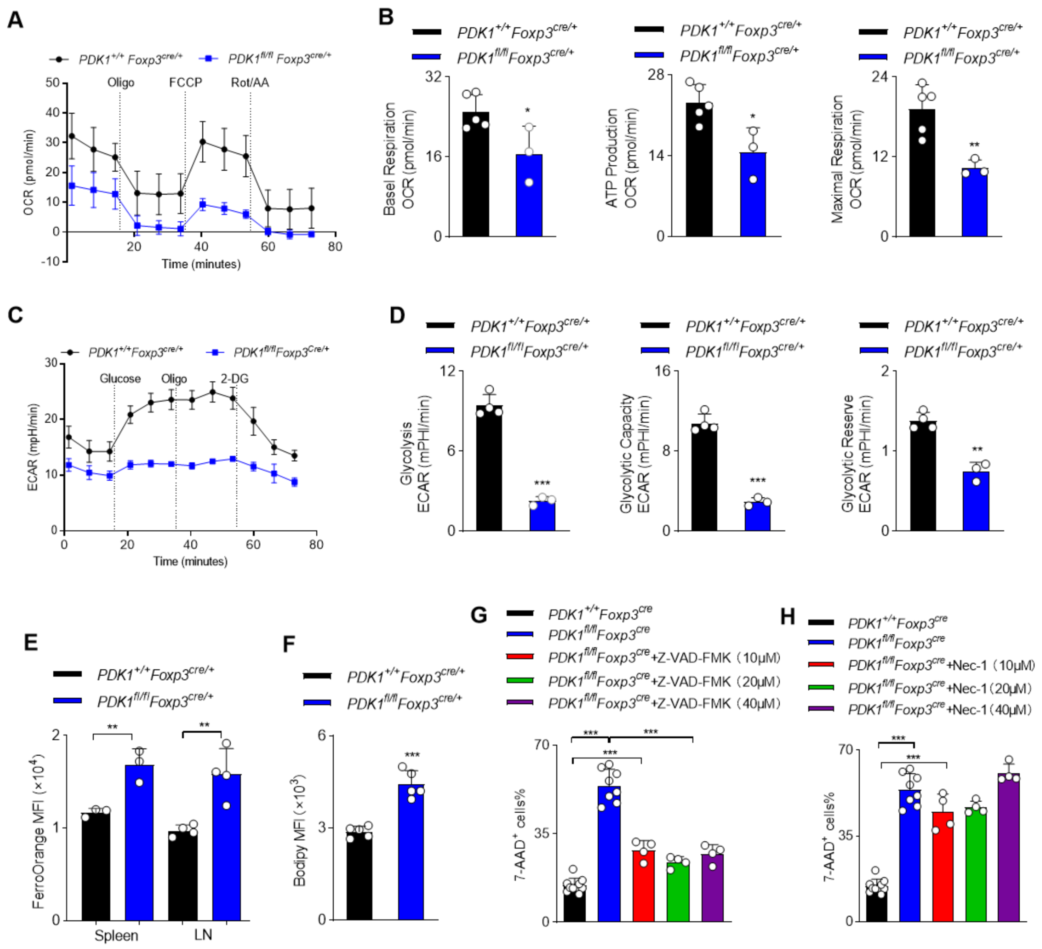

Figure S6. Treg cells require PDK1 to inhibit its apoptosis and Iron-dependent cell death. (A, B) OCR analysis of Treg sorted form spleen and lymph nodes (LN) of $PDK1^{+/+}Foxp3^{Cre/+}$ and $PDK1^{fl/fl}Foxp3^{Cre/+}$ mice, statistics of 3-5 duplicates were shown in **B**. **(C, D)** ECAR analysis of Treg sorted form spleen and lymph nodes (LN) of $PDK1^{+/+}Foxp3^{Cre/+}$ and $PDK1^{fl/fl}Foxp3^{Cre/+}$ mice, statistics of 3-4 duplicates were

shown in **D**. **(E)** MFI statistic of Fe^{2+} level in Treg cells from spleen and lymph nodes (LN) of *PDK1*$^{+/+}$*Foxp3*$^{Cre/+}$ and *PDK1*$^{fl/fl}$*Foxp3*$^{Cre/+}$ mice (n⩾3). **(F)** MFI statistic of Lipid ROS level in Treg cells from *PDK1*$^{+/+}$*Foxp3*$^{Cre/+}$ and *PDK1*$^{fl/fl}$*Foxp3*$^{Cre/+}$ mice (n=5). **(G, H)** Treg cells from *PDK1*$^{+/+}$*Foxp3*^{Cre} and *PDK1*$^{fl/fl}$*Foxp3*^{Cre} mice were treated with or without different concentrations of Z-VAD-FMK and Nec-1 for 24h, cell viability analyzed using 7-AAD, statistics of 4-10 duplicates were shown. $^{*}P \le 0.05$; $^{**}P \le 0.01$; $^{***}P \le 0.001$; unpaired Student's t test. Data represent three independent experiments.

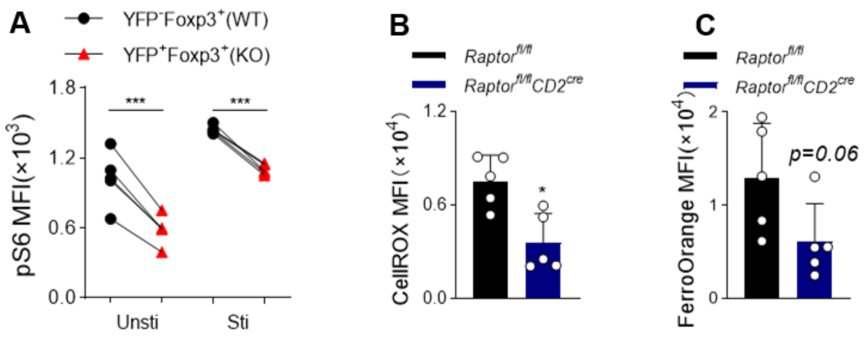

Figure S7. PDK1 regulates iron homeostasis of Treg cells independent of mTORC1 signaling. **(A)** Phosphorylation of p-S6 in $CD4^{+}$ $Foxp3^{+}YFP^{-}$ T cells (WT) and $CD4^{+}$ $Foxp3^{+}YFP^{+}$ T cells (KO) from *PDK1*$^{fl/fl}$*Foxp3*$^{Cre/+}$ mice freshly detected and activated with anti-CD3/CD28 for 30 min. (Paired Student's t test) Unsti: unstimulated, Sti: stimulated (n=5). **(B)** MFI statistic of total ROS level in $CD4^{+}CD25^{+}$ Treg cells from *Raptor*$^{fl/fl}$*CD2*^{Cre} and their control mice (n=5). **(C)** MFI statistic of Fe^{2+} level in

$CD4^+CD25^+$ Treg cells from *Raptor*$^{fl/fl}$*CD2*^{Cre} and their control mice (n=5). $^{*}P \leq 0.01$; $^{***}P \leq 0.001$; unpaired Student's t test. Data represent two independent experiments.
